# Deep single-cell RNA-seq data clustering with graph prototypical contrastive learning

**DOI:** 10.1093/bioinformatics/btad342

**Published:** 2023-05-26

**Authors:** Junseok Lee, Sungwon Kim, Dongmin Hyun, Namkyeong Lee, Yejin Kim, Chanyoung Park

**Affiliations:** Department of Industrial and Systems Engineering, KAIST, Daejeon 34141, Republic of Korea; Department of Industrial and Systems Engineering, KAIST, Daejeon 34141, Republic of Korea; Institute of Artificial Intelligence, POSTECH, Pohang 37673, Republic of Korea; Department of Industrial and Systems Engineering, KAIST, Daejeon 34141, Republic of Korea; Center for Safe Artificial Intelligence for Healthcare, School of Biomedical Informatics, University of Texas Health Science Center at Houston, Houston, TX 77030, United States; Department of Industrial and Systems Engineering, KAIST, Daejeon 34141, Republic of Korea

## Abstract

**Motivation:**

Single-cell RNA sequencing enables researchers to study cellular heterogeneity at single-cell level. To this end, identifying cell types of cells with clustering techniques becomes an important task for downstream analysis. However, challenges of scRNA-seq data such as pervasive dropout phenomena hinder obtaining robust clustering outputs. Although existing studies try to alleviate these problems, they fall short of fully leveraging the relationship information and mainly rely on reconstruction-based losses that highly depend on the data quality, which is sometimes noisy.

**Results:**

This work proposes a graph-based prototypical contrastive learning method, named scGPCL. Specifically, scGPCL encodes the cell representations using Graph Neural Networks on cell–gene graph that captures the relational information inherent in scRNA-seq data and introduces prototypical contrastive learning to learn cell representations by pushing apart semantically dissimilar pairs and pulling together similar ones. Through extensive experiments on both simulated and real scRNA-seq data, we demonstrate the effectiveness and efficiency of scGPCL.

**Availability and implementation:**

Code is available at https://github.com/Junseok0207/scGPCL.

## 1 Introduction

By measuring transcriptome-wide gene expression at single cell level, single-cell RNA sequencing (scRNA-seq) studies have helped researchers to better understand complex biological questions, such as exploring cellular heterogeneity (Shapiro *et al.* 2013, Kolodziejczyk *et al.* 2015). To this end, clustering techniques that identify cell types of cells have been widely studied. Early studies mainly relied on dimensionality reduction techniques, such as PCA, t-SNE (Maaten 2008), and UMAP (McInnes *et al.* 2018), to obtain representative cell representations, which is then followed by traditional clustering algorithms (e.g. k-means clustering) to identify cell types. Although these methods have been shown to be effective on bulk RNA-seq and microarray data, they fall short of handling scRNA-seq data that typically contains tens of thousands dimensional features, which incurs the curse of dimensionality leading to poor clustering performance of traditional clustering algorithms. Moreover, a considerable fraction of truly expressed genes is not well observed in scRNA-seq data owing to the pervasive dropout phenomenon, which results in false zero counts incurring further difficulties in analyzing scRNA-seq data.

Recently, various methods have been proposed to address the aforementioned difficulties of clustering cells in the scRNA-seq data. To tackle the limitation incurred by the curse of dimensionality inherent in the similarities calculated based on the commonly used Euclidean distance, SNN-Cliq (Xu and Su 2015) proposes a shared nearest neighbor-based similarity. Moreover, SIMLR (Wang *et al.* 2017) proposes to learn cell-to-cell similarity via multiple kernel learning. Besides, CIDR (Lin *et al.* 2017) first calculates the dissimilarity matrix using imputed gene expression, and then performs principal coordinate analysis (PCoA) to alleviate the impact of dropouts. However, due to the lack of expressive power of the models, these methods do not generalize well in the scRNA-seq data across various experiments and platforms (Tian *et al.* 2019, Ciortan and Defrance 2021, Wang *et al.* 2021, Wan *et al.* 2022).

In the last few years, Deep Neural Networks (DNN) have emerged as powerful feature extractors for dimensionality reduction or clustering, and several recent methods have translated this success to scRNA-seq data. Most of them leverage an autoencoder network that learns cell representations with compression and reconstruction scheme. Specifically, DCA (Eraslan *et al.* 2019) fits the zero-inflated negative binomial (ZINB) distribution with an autoencoder by replacing the conventional mean squared error (MSE) loss with the ZINB negative log-likelihood. Inspired by the well-known generative model VAE (Kingma and Welling 2013), SCVIS (Ding *et al.* 2018) adopts a generative approach to learn cell representations with t-SNE regularization to preserve the local structure. Although DCA and SCVIS have shown to be effective for characterizing scRNA-seq data, they mainly focus on the imputation and dimensionality reduction tasks for scRNA-seq data, respectively, and do not particularly consider the cell clustering task during the training process. On the other hand, scDeepCluster (Tian *et al.* 2019) succeeds in enhancing the clustering performance by fine-tuning DCA based on a clustering loss aiming at directly optimizing the cell representations for clustering. However, as scDeepCluster does not leverage any relational information between cells, it is hard to learn cell representations for accurate clustering when input features are not informative enough (e.g. input gene expression matrix is highly sparse).

Although the reconstruction-based representation learning is a dominant way of learning cell representations in scRNA-seq data (Tian *et al.* 2019, Chen *et al.* 2020a, Wang *et al.* 2021, Gan *et al.* 2022), contrastive learning-based representation learning methods (Oord *et al.* 2018, Chen *et al.* 2020b, He *et al.* 2020) have also been investigated for the scRNA-seq data (Ciortan and Defrance 2021, Wan *et al.* 2022). In natural language processing (NLP) and computer vision (CV) domains, contrastive learning has emerged as an effective way to learn word or image representations, and it has been shown to outperform autoencoder-based (i.e. reconstruction-based) approaches in many cases. The main idea of contrastive learning is to push apart semantically dissimilar pairs and pull together similar pairs (e.g. augmented views of the same image) in the representation space. The success of contrastive learning has also been transferred to the graph domain, and it has been shown that contrastive learning-based methods, such as GRACE (Zhu *et al.* 2020) and GCA (Zhu *et al.* 2021), outperform reconstruction-based methods in many cases. We argue that the blackcontrastive learning framework is especially well-suited for analyzing highly sparse scRNA-seq data, since contrastive learning generates cell representations that are more tolerant to noise than the reconstruction-based representation learning framework. This is because the contrastive learning framework aims to learn cell representations by comparing the similarity between a positive pair (e.g. augmented views of the same cell) and negative pairs in the representation space, whereas the reconstruction-based approaches solely rely on reconstructing the input matrix that is highly sparse in nature due to the inevitable dropout phenomenon. Recently proposed contrastive-sc (Ciortan and Defrance 2021) adopts instance-wise contrastive learning where a positive pair is defined by randomly masking some gene expression values of a cell, while all other cells are considered as negative pairs. However, we argue that such a simple augmentation scheme is less sufficient to fully leverage the benefit of contrastive learning as it fails to incorporate any relational information between cells. Inspired by VIME (Yoon *et al.* 2020), scNAME (Wan *et al.* 2022) proposes an augmentation scheme that combines masked and shuffled features and estimates the mask matrix from the augmented view and neighborhood contrastive loss that enhances similarity between *k*-nearest neighbors. However, it still overlooks the relational information inherent in scRNA-seq data, and does not use contrastive learning on the pre-training phase that is an important step for learning cell representations.

To leverage the relational information between cells, several existing studies construct a cell–cell graph on which Graph Neural Networks (GNNs) are applied. Specifically, scGNN (Wang *et al.* 2021) constructs a cell–cell graph based on the learned cell representations to capture the relational information between cells, and uses cell type-specific cluster autoencoders to help discover cell type-specific information. Moreover, scDSC (Gan *et al.* 2022) enhances scDeepCluster by introducing a cell–cell graph encoded with GNNs to better capture the relationship between cells. Although the existing studies have shown the effectiveness of reflecting the relational information between cells for the cell clustering task, we argue that the inherent sparseness of the input gene expression matrix used to compute the similarity between cells leads to a low-quality cell–cell graph, which eventually hinders the construction of high-quality cell representations. (The experiments about this argument can be found in Supplementary Fig. S1 with a detailed description in Supplementary Notes S1.3.1.)

In this article, we propose a graph-based prototypical contrastive learning method aiming at clustering cells in the scRNA-seq data that fully leverages the relational information between cells. Different from existing studies that construct a cell–cell graph by connecting similar cells based on the pre-calculated cell–cell similarities, we introduce a *bipartite cell–gene graph*, which is constructed by connecting two nodes (i.e. cell and gene), if a particular gene is expressed in that cell on the given input gene expression matrix. We argue that since the proposed cell–gene graph preserves the original gene expression values in the given gene expression matrix rather than calculating the cell–cell similarity, it preserves the natural relationship inherent in the given data, which eventually maintains the quality of the constructed graph.

Moreover, we augment the graph by sampling subgraphs and masking features of the bipartite cell–gene graph, and generate two differently augmented views from the original graph. Then, we conduct instance-wise contrastive learning by defining a positive pair as the differently augmented views of the same cell, while treating all other cells as negatives. However, the instance-wise contrastive learning may incur an adverse effect on the clustering task at hand due to the sampling bias (Chuang *et al.* 2020) caused by incorrect negative pairs (i.e. considering a pair of cells in the same cell type as a negative pair). To tackle this problem, inspired by PCL (Li *et al.* 2020), we propose to adopt the prototypical contrastive learning scheme to help our model learn cluster (i.e. cell type) specific information and alleviate the sampling bias by pulling together an anchor cell and its corresponding cluster prototype. Hence, we name our proposed method as single-cell Graph Prototypical Contrastive Learning (scGPCL). Through extensive experiments on both simulated and real scRNA-seq data, we demonstrate the robustness and efficacy of scGPCL compared with state-of-the art methods.

## 2 Materials and methods

### 2.1 Data preprocessing

We follow the preprocessing step used in the DCA and scDeepCluster. We conduct data preprocessing using SCANPY (Wolf *et al.* 2018) packages. Specifically, given the raw read count matrix $X^{\mathrm{count}}$ (i.e. gene expression matrix), we calculate the library size of each cell, *l_i_*, as the total number of read counts per cell and obtain the size factor of each cell, *s_i_*, by dividing library size *l_i_* by the median of the library sizes. We then obtain the normalized read count *x* by dividing the read count by the size factor of each cell followed by log(x+1) transformation. We also define a bipartite cell–gene graph denoted by G=(V,E), where $V=\left\{V_{c},V_{g}\right\}$ represents the set of nodes, where *V_c_* and *V_g_* denote the set of cell nodes ($|V_{c}|=N_{c}$) and gene nodes ($|V_{g}|=N_{g}$), respectively. Moreover, E represents the set of edges, where a cell node and a gene node are connected with an associated expression value (i.e. edge weight) if the gene is expressed in that cell. As for the cell node features denoted by $X_{c}\in R^{N_{c}\times N_{g}}$, we further conduct standard scaling to make the features of each cell node have zero mean and unit variance. As for the gene node features denoted by $X_{g}\in R^{N_{g}\times F}$, we set them with randomly initialized learnable features. Note that scGPCL leverages the cell–gene bipartite graph obtained from the original gene expression matrix to preserve the natural relationship between cells inherent in the given data rather than leveraging a cell–cell graph constructed based on the pre-calculated cell–cell similarity as it may incur information loss if the constructed graph is not proper.

### 2.2 Proposed method: scGPCL

**Figure 1.**
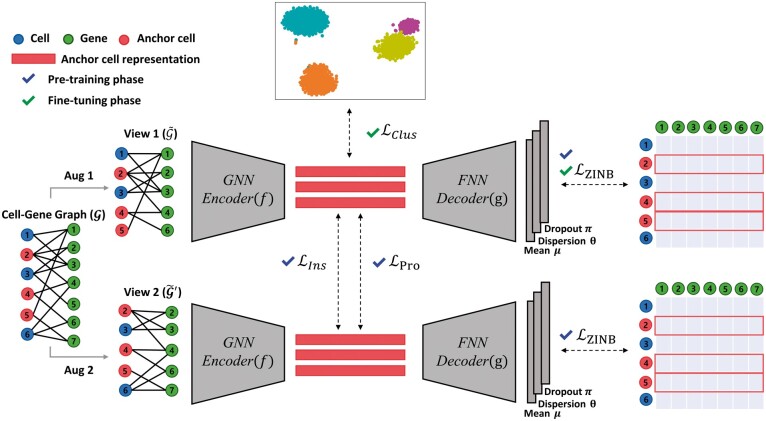
The overall architecture of scGPCL. Given a cell–gene Graph G, scGPCL augments G into two graphs $\tilde{G}$ and $\tilde{G^{\prime }}$, and obtains anchor cell representations $\tilde{H}$ and ${\tilde{H}}^{\prime }$ by passing them through a shared GNN encoder *f*. Then, using these anchor cell representations, scGPCL reconstructs the gene expression matrix by maximizing ZINB likelihood (i.e. $L_{\text{ZINB}}$), while also updating them by using contrastive (i.e. $L_{\text{Ins}}$) and prototypical (i.e. $L_{\text{Pro}}$) losses of the anchor cells. After that, scGPCL finetunes the cell representations with the clustering loss (i.e. $L_{\text{Clus}}$) to explicitly optimize for the clustering task and utilize ZINB loss (i.e. $L_{\text{ZINB}}$) to preserve the local structure of data.

scGPCL is a graph-based prototypical contrastive learning method designed for clustering cells in the scRNA-seq data, and its overall architecture is shown in Fig. 1. Specifically, the learning strategy of scGPCL is divided into the pre-training (Section 2.2.1) and fine-tuning phases (Section 2.2.2). In the pre-training phase, we generate two augmented views of the original cell–gene graph by applying two different stochastic augmentation functions composed of subgraph sampling and feature masking. It is worth mentioning that due to technical limitations in sequencing, only a fraction of the gene expression is detected for each cell. Motivated by the nature of scRNA-seq data, we create augmented views using subgraph sampling and feature masking that drop a fraction of gene expression in cell–gene data. Next, we encode the augmented graphs by passing them through a GNN encoder to obtain the representations for the cells in the current batch. Then, scGPCL learns cell representations by not only relying on the reconstruction-based loss (i.e. estimating ZINB distribution using decoder layer, $L_{\text{ZINB}}$), but also leveraging the instance-wise (i.e. $L_{\text{Ins}}$) and prototypical (i.e. $L_{\text{Pro}}$) contrastive losses. As shown in Supplementary Fig. S2, scGPCL randomly selects some cell nodes for training (i.e. anchor nodes), and then defines the positive pairs from both the instance-wise and the prototypical relation. More precisely, the instance-wise positive pair is defined by the cell representations of a certain cell obtained from the two views, whereas the prototypical positive pair is defined by the cell representations of a cell from one view (i.e. view 1) and the cluster prototype assigned for the cell from the other view (i.e. view 2). All other instances and prototypes are used to construct the negative pairs. Furthermore, we adopt the fine-tune strategy following existing studies (Guo *et al.* 2017, Tian *et al.* 2019) to improve the clustering performance. In the fine-tuning phase, we exploit the cluster loss (i.e. $L_{\text{Cluster}}$) to explicitly optimize the anchor cell representations using a self-training strategy for clustering task, and maintain the reconstruction-based loss (i.e. $L_{\text{ZINB}}$) to preserve the local structure of data. In the following sections, we explain the training strategy in more detail.

#### 2.2.1 Phase 1: pre-training

To begin with, we define a stochastic augmentation through a composition of subgraph sampling and feature masking, and generate two augmented graphs denoted as $\tilde{G}=(\tilde{V},\tilde{E})$ and $\tilde{G^{\prime }}=({\tilde{V}}^{\prime },{\tilde{E}}^{\prime })$. More precisely, we sample subgraphs from the original cell–gene bipartite graph G by using HGSampling technique (Hu *et al.* 2020), and mask some features of both *X_c_* and *X_g_*. Then, given *N_b_* anchor nodes in the current batch (Note that we randomly select *N_b_* nodes in each iteration, and we make sure all *N_c_* nodes are covered in each epoch. That is, we run $\left\lceil \frac{N_{c}}{N_{b}}\right\rceil$ iterations in each epoch.), scGPCL obtains anchor cell representations $\tilde{H}=f(\tilde{G})\in R^{N_{b}\times d}$ and ${\tilde{H}}^{\prime }=f({\tilde{G}}^{\prime })\in R^{N_{b}\times d}$ from two differently augmented graphs by passing them through a GNN encoder *f*, where *d* is the dimensionality of the cell representation. More precisely, we leverage the GraphSAGE (Hamilton *et al.* 2017) encoder as our backbone whose aggregation scheme of each layer is given as follows: where Mean(·) is the average of the input vectors, $N(c_{i})$ is the neighboring nodes of cell *i*, and $N(g_{i})$ is the neighboring nodes of gene *i*. Moreover, $h_{c_{i}}^{\left(k\right)}\in R^{d_{c}^{\left(k\right)}}$ and $h_{g_{i}}^{\left(k\right)}\in R^{d_{g}^{\left(k\right)}}$ are the hidden representations of the cell *i* and gene *i* at the *k*th layer, respectively, and $d_{c}^{\left(k\right)}$ and $d_{g}^{\left(k\right)}$ denote the dimensionality of the cell and gene representations at the *k*th layer, respectively. We introduce trainable parameters for cell nodes, i.e. $W_{c}^{\left(k\right)}\in R^{d_{c}^{\left(k+1\right)}\times d_{c}^{\left(k\right)}}$ and $W_{c}^{\prime (k)}\in R^{d_{c}^{\left(k+1\right)}\times d_{c}^{\left(k\right)}}$ each of which denotes the weight matrices for transforming neighboring cell nodes and the center cell node itself (i.e. self-loop), respectively. Likewise, we introduce trainable parameters for gene nodes, i.e. $W_{g}^{\left(k\right)}\in R^{d_{g}^{\left(k+1\right)}\times d_{g}^{\left(k\right)}}$ and $W_{g}^{\prime (k)}\in R^{d_{g}^{\left(k+1\right)}\times d_{g}^{\left(k\right)}}$ each of which denotes the weight matrices for transforming neighboring gene nodes and the center gene node itself (i.e. self-loop), respectively. Note that except for the raw input features, i.e. $d_{c}^{\left(0\right)}$ and $d_{g}^{\left(0\right)}$, the dimensionality for the cell and gene representations is same.


(1) $$\{\begin{array}{c} h_{c_{i}}^{\left(k+1\right)}=W_{c}^{\prime (k)}h_{c_{i}}^{\left(k\right)}+W_{g}^{\left(k\right)}\mathrm{Mea}n_{g_{j}\in N(c_{i})}(h_{g_{j}}^{\left(k\right)})\\ h_{g_{i}}^{\left(k+1\right)}=W_{g}^{\prime (k)}h_{g_{i}}^{\left(k\right)}+W_{c}^{\left(k\right)}\mathrm{Mea}n_{c_{j}\in N(g_{i})}(h_{c_{j}}^{\left(k\right)}) \end{array}$$

**Instance-wise contrastive loss.** After the encoding process described above, we apply the contrastive learning framework whose overview can be found in Supplementary Fig. S2. More precisely, scGPCL computes the infoNCE (Oord *et al.* 2018) objective for each positive cell node pair $\left({\tilde{h}}_{i},{\tilde{h}}_{i}^{\prime }\right)$, where ${\tilde{h}}_{i}$ and ${\tilde{h}}_{i}^{\prime }$ are the *i*th row of the $\tilde{H}$ and ${\tilde{H}}^{\prime }$, respectively, which denote the representation of cell *i* from the two views: where $l_{\text{Ins}}(\cdot ,\cdot )$ is infoNCE loss, sim(·,·) is the cosine similarity between two vectors, $1_{\left[\cdot \right]}$ is the indicator function, and *τ* is the temperature hyperparamter. We use the two representations of the same cell obtained from two augmented views as the positive pair (i.e. ${\tilde{h}}_{i}$ and ${\tilde{h}}_{i}^{\prime }$), and all others are considered as negative pairs. The overall instance-wise contrastive loss is given by:


(2) $$l_{\text{Ins}}({\tilde{h}}_{i},{\tilde{h}}_{i}^{\prime })=\text{log}\;\frac{e^{\left(\mathrm{sim}({\tilde{h}}_{i},{\tilde{h}}_{i}^{\prime })/\tau \right)}}{\sum_{j=1}^{N_{b}}\;1_{\left[i\neq j\right]}e^{\left(\mathrm{sim}({\tilde{h}}_{i},{\tilde{h}}_{j})/\tau \right)}+\sum_{j=1}^{N_{b}}e^{\left(\mathrm{sim}({\tilde{h}}_{i},{\tilde{h}}_{j}^{\prime })/\tau \right)}}$$

(3) $$L_{\text{Ins}}=-\frac{1}{2N_{b}}\sum_{i=1}^{N_{b}}\;[l_{\text{Ins}}({\tilde{h}}_{i},{{\tilde{h}}^{\prime }}_{i})+l_{\text{Ins}}({{\tilde{h}}^{\prime }}_{i},{\tilde{h}}_{i})].$$

By minimizing the above contrastive loss, scGPCL learns the cell representations by pulling together positive pairs and pushing apart negative pairs in the cell representation space. Note that such a contrastive learning scheme is especially beneficial for scRNA-seq data, because it is hard to learn cell representations with only reconstruction-based loss when the given input matrix is highly sparse due to the pervasive dropout phenomenon.

**Prototypical contrastive learning framework.** However, the instance-wise contrastive loss exhibits an inherent limitation, called sampling bias (Chuang *et al.* 2020). In other words, given an anchor cell, since all other cells apart from the augmented version of the anchor cell are considered as negative instances, it is highly likely that negative instances contain cells that belong to the same cell type as the anchor cell, which is undesirably pushed apart from the anchor cell. To alleviate this problem, scGPCL adopts the prototypical contrastive learning framework as illustrated in Supplementary Fig. S2. Specifically, we compute cluster prototypes (i.e. cluster centroids) by performing *K*-means clustering on the cell representations obtained from the view 2 (i.e. ${\tilde{H}}^{\prime }$). [Note that using view 1 (i.e. $\tilde{H}$) yields similar performance.] We then treat the pairs of cells assigned to the same prototype as positive pairs, and the remaining pairs as negative pairs. scGPCL performs this clustering process *T* times with different *K*s to find semantically similar groups across various granularities. The loss for a particular cell *i* is given as follows: where *K_t_* denotes the number of prototypes in *t*th iteration, and $z_{s}^{t}\in R^{d}$ denotes the representation of the prototype *s* in the *t*th iteration. The indicator function $1_{\left({\tilde{h}}_{i}\in z_{s}^{t}\right)}$ is defined as 1 if the cell *i* belongs to the cluster represented by $z_{s}^{t}$, and 0 otherwise. Note that the prototypical loss defined as above is especially beneficial for clustering task, because it groups cells that belong to the same cell type together by minimizing the distance between each cell and the corresponding cluster prototype. The overall prototypical contrastive loss is given as follows:


(4) $$l_{\text{Pro}}({\tilde{h}}_{i})=\frac{1}{T}\sum_{t=1}^{T}\sum_{s=1}^{K_{t}}1_{\left({\tilde{h}}_{i}\in z_{s}^{t}\right)}\;\text{log}\;\frac{e^{\left(\mathrm{sim}({\tilde{h}}_{i},z_{s}^{t})/\tau \right)}}{\sum_{j=1}^{K_{t}}e^{\left(\mathrm{sim}({\tilde{h}}_{i},z_{j}^{t})/\tau \right)}}$$

(5) $$L_{\text{Pro}}=-\frac{1}{N_{b}}\sum_{i=1}^{N_{b}}l_{\text{Pro}}({\tilde{h}}_{i}).$$

**ZINB-based reconstruction loss.** Following existing studies (Eraslan *et al.* 2019, Tian *et al.* 2019, Gan *et al.* 2022), we assume that the gene expression matrix follows the ZINB distribution to capture the characteristic of the scRNA-seq data. Specifically, the ZINB distribution is defined as: where *μ*, *θ*, and *π* represent the parameters of ZINB distribution that are mean, dispersion, and dropout probability, respectively. To estimate these parameters, we introduce a shared feed-forward decoder layer *g*, and an additional layer for each of the three parameters. Specifically, the output of the decoder $D=g(H)\in R^{N_{b}\times N_{g}}$ is independently fed into additional layers for three parameters (i.e. *μ*, *θ*, and *π*) as follows: where $S\in R^{N_{b}\times N_{b}}$ is a diagonal matrix whose diagonal element for the *i*th row is the size factor (i.e. *s_i_*) of cell *i*, $M\in R^{N_{b}\times N_{g}},\;\Theta \in R^{N_{b}\times N_{g}}$, and $\Pi \in R^{N_{b}\times N_{g}}$ are the matrix representation of estimated mean, dispersion, and dropout probability, respectively, and $W_{\mu }\in R^{N_{g}\times N_{g}},\;W_{\theta }\in R^{N_{g}\times N_{g}}$, and $W_{\pi }\in R^{N_{g}\times N_{g}}$ are trainable parameters. Note that the exponential function is adopted for *M* and Θ due to the non-negative range of mean and dispersion, whereas the sigmoid function is adopted for Π as the dropout probability lies between 0 and 1. The ZINB-based reconstruction loss for the estimated parameters given by Equation 7 is calculated based on the negative log-likehlihood of ZINB distribution as follows: where $X^{\mathrm{count}}$ denotes the raw read count matrix, and $X_{ij}^{\mathrm{count}}$, Π_*ij*_, *M_ij_*, and Θ_*ij*_ denote the element at the *i*th row and the *j*th column for each matrix. The overall ZINB-based reconstruction loss of scGPCL from the two views is given by: where $\tilde{\Pi },\;\tilde{M}$, and $\tilde{\Theta }$ represent the estimated parameters from the view 1, and ${\tilde{\Pi }}^{\prime },\;{\tilde{M}}^{\prime },\;{\tilde{\Theta }}^{\prime }$ denote those from the view 2.


(6) $$\begin{array}{c} NB(x|\mu ,\theta )=\frac{\Gamma (x+\theta )}{x!\Gamma (\theta )}{\left(\frac{\theta }{\theta +\mu }\right)}^{\theta }{\left(\frac{\mu }{\theta +\mu }\right)}^{x}\\ \mathrm{ZINB}(x|\pi ,\mu ,\theta )=\pi \delta_{0}(x)+(1-\pi )NB(x|\mu ,\theta ) \end{array}$$

(7) $$M=S\times \text{exp}(DW_{\mu }),\Theta =\text{exp}(DW_{\theta }),\Pi =\text{sigmoid}(DW_{\pi })$$

(8) $$\begin{array}{c} l_{\text{ZINB}}(\Pi ,M,\Theta )=\\ \frac{1}{N_{b}\times N_{g}}\sum_{i=1}^{N_{b}}\sum_{j=1}^{N_{g}}-\;\text{log}(\text{ZINB}(X_{ij}^{\mathrm{count}}|\Pi_{ij},M_{ij},\Theta_{ij})) \end{array}$$

(9) $$L_{\text{ZINB}}^{\text{Pre}}=\frac{1}{2}[l_{\text{ZINB}}(\tilde{\Pi },\tilde{M},\tilde{\Theta })+l_{\text{ZINB}}({\tilde{\Pi }}^{\prime },{\tilde{M}}^{\prime },{\tilde{\Theta }}^{\prime })]$$

**Final objectives of pre-training phase.** Finally, scGPCL combines $L_{\text{Ins}},\;L_{\text{Pro}}$, and $L_{\text{ZINB}}^{\text{Fine}}$ with balance coefficients *λ*_1_ and *λ*_2_ to learn cell representations in the pre-training phase as follows:


(10) $$L_{\text{Pre}}=\lambda_{1}L_{\text{Ins}}+\lambda_{2}L_{\text{Pro}}+L_{\text{ZINB}}^{\text{Pre}}$$

Throughout all the experiments, we set *λ*_2_ 20 times smaller than that of *λ*_1_ (i.e. $\lambda_{2}=0.05$ and $\lambda_{1}=1.0$) because putting too much emphasis on the prototypical contrastive loss (i.e. $L_{\text{Pro}}$) may incur a negative effect, called confirmation bias (Arazo *et al.* 2020). That is, if a certain cell is initially assigned to an incorrect cluster, its representation would be forced to be close to cells that belong to the cluster, and this aggravates if *λ*_2_ is large.

During the pre-training phase, we define the convergence of scGPCL based on change of cluster assignments from two consecutive epochs. More specifically, we stop training if ARI between cluster assignments from two consecutive epochs is higher than pre-defined threshold *r*. The overall architecture for scGPCL is depicted in Fig. 1, and we further provide the pseudo-code of pre-training phase of scGPCL in the Supplementary Algorithm S1.

#### 2.2.2 Phase 2: fine-tuning

**Clustering task-oriented loss.** In the fine-tuning phase, scGPCL adopts self-training (Zhou *et al.* 2022) that encourages each cell to be assigned to a cluster of high confidence. More precisely, given a soft cluster assignment distribution matrix $Q\in R^{N_{b}\times K}$ whose each row denotes the soft cluster assignment distribution of each cell, we introduce a target distribution matrix $P\in R^{N_{b}\times K}$ that is obtained by sharpening *Q*, and minimize the Kullback-Leibler (KL) divergence between the two distributions as follows: where *q_ik_* and *p_ik_* are the assignment probabilities of cell *i* to cluster *k* in terms of the soft cluster assignment distribution matrix *Q* and the target distribution matrix *P*. Note that *q_ik_* is calculated by measuring the similarity between the representation of cell *i*, (i.e. *h_i_*), and the centroid of cell *i*, (i.e. *c_k_*), based on the Student’s *t*-distribution as follows: where *α* is the degree of freedom of the Student’s *t*-distribution. Then, the target distribution *p_ik_* is calculated by normalizing the second power of the soft assignment distribution by the frequency per cluster as follows: where $f_{k}=\sum_{i=1}^{N_{b}}q_{ik}$ is the soft cluster frequencies used to prevent degenerate solutions in which case some clusters are not assigned any instances at all. In other words, Equation 13 sharpens *q_ik_* (i.e. *p_ik_* is a sharpened version of *q_ik_*), by making a large value to be larger and a small value to be smaller. As a result, by minimizing the KL divergence defined in Equation 11, in which *Q* and *P* are defined as in Equations 12 and 13, respectively, we aim to provide more confident cluster assignments to each cell, which in turn explicitly optimizes the cell representations for the cell clustering task. Note that we run *K*-means clustering only once before starting the fine-tuning phase to initialize the cluster centroids (i.e. ${\left\{c_{k}\right\}}_{k=1}^{K}$), and *K*-means clustering is not performed anymore thereafter. Moreover, to improve the robustness of the clustering assignments, we use $\tilde{H}$ obtained from an augmented graph $\tilde{G}$ (i.e. $\tilde{H}=f(\tilde{G})$) to compute the soft cluster assignment matrix $Q\in R^{N_{b}\times K}$.


(11) $$L_{\text{Cluster}}=D_{\text{KL}}(P∥Q)=\sum_{i=1}^{N_{b}}\sum_{k=1}^{K}p_{ik}\;\text{log}\;\frac{p_{ik}}{q_{ik}}$$

(12) $$q_{ik}=\frac{{\left(1+||h_{i}-c_{k}|{|}_{2}^{2}/\alpha \right)}^{-\frac{\alpha +1}{2}}}{\sum_{j=1}^{K}{\left(1+||h_{i}-c_{j}|{|}_{2}^{2}/\alpha \right)}^{-\frac{\alpha +1}{2}}}$$

(13) $$p_{ik}=\frac{q_{ik}^{2}/f_{k}}{\sum_{j=1}^{K}q_{ij}^{2}/f_{j}}$$

**Final objectives of fine-tuning phase.** Finally, the overall loss of scGPCL in the fine-tuning phase is defined by combining $L_{\text{Cluster}}$ and $L_{\text{ZINB}}^{\text{Fine}}$ with a balance coefficient *λ*_3_ as follows:


(14) $$L_{\text{Fine}}=L_{\text{Cluster}}+\lambda_{3}L_{\text{ZINB}}^{\text{Fine}}$$

Note that scGPCL maintains the reconstruction-based loss (i.e. $L_{\text{ZINB}}^{\text{Fine}}$) during the fine-tuning phase to preserve the local structure of data. Note that as the contrastive loss is not involved in the fine-tuning phase, we only generate a single augmented view, i.e. $\tilde{G}$, on which the ZINB loss is defined as follows:


(15) $$L_{\text{ZINB}}^{\text{Fine}}=l_{\text{ZINB}}(\tilde{\Pi },\tilde{M},\tilde{\Theta })$$

We perform the fine-tuning phase until the change of cluster assignment between two consecutive epochs is less than a threshold, i.e. tol%. The pseudo-code for the fine-tuning phase can also be found in Supplementary Algorithm S2.

### 2.3 Statistical test

To verify the superiority of scGPCL over existing methods, we conduct a one-sided paired t-test for all our experiments in terms of normalized mutual information (NMI), and report the corresponding *P*-value. More precisely, for experiments where scGPCL did not outperform other method, we define the null hypothesis as $H_{0}:NMI_{\text{baseline}}>NMI_{\mathrm{scGPCL}}$, and for experiments where scGPCL we define the null hypothesis as $H_{0}:NMI_{\mathrm{scGPCL}}>NMI_{\text{baseline}}$. In addition, we also conduct one-sided Wilcoxon signed ranks test (Demšar 2006) for real scRNA-seq datasets to demonstrate the effectiveness of scGPCL in real-world data.

## 3 Results

### 3.1 Evaluation of scGPCL on simulated data

To demonstrate the effectiveness of scGPCL, we simulate scRNA-seq data with Splatter (Zappia *et al.* 2017) package assuming three situations in which learning cell representations may be challenging: **Case 1:** Gene expression matrix is highly sparse due to the dropout phenomena (Section 3.1.1), **Case 2:** Gene expression values contain relatively low signal strength required for clustering (Section 3.1.2), and **Case 3:** The size of cell clusters is imbalanced in number (Section 3.1.3). To evaluate the clustering performance, we compare scGPCL with seven state-of-the art baselines using three standard clustering evaluation metrics, i.e. normalized mutual information (NMI), clustering accuracy (CA), and adjusted rand index (ARI) and we replace CA with Macro-F1 and Micro-F1 score both of which are well suited for the imbalance cases in **Case 3**. All these metrics measure the concordance between the ground truth cluster assignments and the predicted cluster assignments, and higher values indicate better performance. Further details about these metrics are described in Supplementary Notes S1.2 and we report the paired t-test results about the following experiments in Supplementary Table S3.

#### 3.1.1 Case 1: evaluation under dropout phenomena

**Figure 2.**
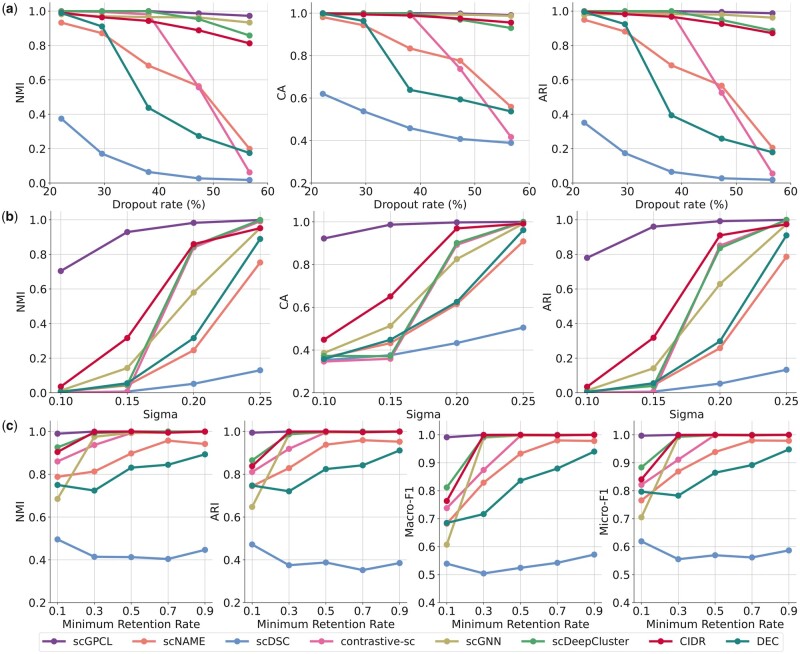
Performance comparisons of scGPCL and other baselines on the simulated dataset. (a), (b), and (c) represent the performance over the various dropout rates, sigmas (small sigma indicates low signals for clustering), and minimum retention rates, respectively.

We analyze the robustness of scGPCL under the dropout phenomena of scRNA-seq data. To this end, we simulate scRNA-seq data by changing the dropout rates, i.e. varying the dropout.mid in Splatter from 0 to 2 (i.e. [0.0, 0.5, 1.0, 1.5, 2.0]), for evaluations under various dropout rates, where a higher value implies a higher dropout rate. More precisely, we fix the number of cells to 3000 containing 3 clusters and the number of genes to 5000, and set the dropout.shape =−1, and de.fracScale =0.3 following the experimental settings of scDeepCluster. We run scGPCL and other baselines with 10 different random seeds, and report the average performance in Fig. 2a. We observe that scGPCL, scGNN, and scDeepCluster consistently outperform other baselines by obtaining accurate cluster assignments regardless of the dropout rates. We note that the superior models (i.e. scGPCL, scGNN, and scDeepCluster) infuse cluster specific information during training. This indicates that incorporating clustering task-specific information during the training process is crucial when the input gene expression matrix suffers from many dropout events. In Supplementary Fig. S3, we also show that scGPCL, scGNN, and scDeepCluster are generally successful in separating the cells according to their clusters regardless of the dropout rates. To further conduct performance comparison on more challenging situations where there exist fewer cells per cluster, we report the performance and visualization result of scGPCL and baseline methods on the simulated dataset with 6 and 10 clusters in Supplementary Figs S3–S6. In this result, we observe that scGPCL can robustly separate the cluster of the cells even if the gene expression matrix suffers from severe dropout phenomena.

#### 3.1.2 Case 2: evaluation under low signal

Second, we assume the case when the gene expression values have low signals for clustering. In the Splatter package, we can vary the strength of the signals of the gene expression values by controlling the sigma parameter (i.e. de.fracScale) of the log-normal distribution that controls the multiplicative differential expression factors. We generate a simulated dataset by varying the value of de.fracScale from 1 to 2.5 (i.e. [1, 1.5, 2, 2.5]), where a low value implies a lower signal. We also set the number of cells to 3000 containing 3 clusters and the number of genes to 5000, and fix the dropout.shape =−1 and dropout.mid =0.0, following the experimental settings of scDeepCluster. Since the input features (i.e. gene expression values) have low signals, Fig. 2b shows that the performance of every method degrades greatly according to the value of sigma except for scGPCL. Specifically, when sigma is 0.1, scGPCL achieves 0.7039, 0.9221, and 0.7800 in terms of NMI, CA, and ARI, respectively, while all other baseline methods perform poorly. Furthermore, when sigma is 0.15, scGPCL greatly outperforms other baselines by achieving 0.9291, 0.9867, and 0.9605 for NMI, CA, and ARI, respectively {i.e. 194%, 52%, and 202% higher than the second best method [i.e. CIDR Lin *et al.* (2017)] in terms of NMI, CA, and ARI, respectively}. We argue that since baseline methods rely on the reconstruction-based loss, which learns cell representations by reconstructing the input gene expression matrix, they fall short of robustly learning cell representations when the input matrix is not informative enough (i.e. low signal). Although contrastive-sc and scNAME leverage the contrastive learning scheme, since they generates views by only augmenting gene expression values and do not incorporate relationship information, they perform poorly when the strength of the signals of the input gene expression values is weak. On the other hand, scGPCL robustly achieves accurate cluster assignments even if the information from the input features is not sufficient, because scGPCL not only depends on the strength of the signal in gene expression data, but also leverages the relational information inherent in cells by using a cell–gene graph along with the contrastive loss in which a positive pair is defined as the representations obtained from differently augmented graph of the same cell. The results are corroborated by the Supplementary Fig. S7 showing that scGPCL consistently succeeds in learning the cell representation space, while other baseline methods are less effective in learning the space as the strength of the signals of the input gene expression values is decreased (i.e. sigma is 0.1). In addition, we also provide the results regarding various strengths of signal for the case when the number of clusters is 10 in Supplementary Fig. S8.

#### 3.1.3 Case 3: evaluation under imbalanced size of cell clusters

Last but not least, we assume an imbalance case where the difference between the number of cells in the majority cluster and that in the minority cluster are imbalanced (Tian *et al.* 2019, Chen *et al.* 2020a; Wan *et al.* 2022). To quantify the degree of imbalance of the scRNA-seq data, we follow the experimental protocol conducted in scDeepCluster, and define the minimum retention rate, which indicates the ratio of the number of cells in the majority cluster to that in the minority cluster. To generate imbalanced data, given 1500 cells, we set the probability of being assigned to 6 clusters with various minimum retention rates. Note that we fix dropout.shape =−1, dropout.mid =0.0, de.fracScale = 0.4, and set the number of genes to 5000. In addition to NMI and ARI used in previous experiments to evaluate the clustering performance, in Fig. 2c, we newly introduce Macro-F1 and Micro-F1 both of which are more suitable metrics compared with accuracy under imbalanced situations. We observe that scGPCL consistently outperforms the baseline methods on all four metrics. Specifically, scGPCL achieves 0.9914 and 0.9969 in terms of Macro-F1 and Micro-F1, respectively, while the second best method (i.e. scDeepCluster) achieves 0.8118 and 0.8836, respectively. Supplementary Fig. S9 also shows that scGPCL and scDeepCluster can separate the cells that belong to the minority cluster (i.e. red points) with only few mingled points, while other baselines fail to do so. This demonstrates that scGPCL obtains accurate cluster assignments regardless of whether a cell belongs to the majority or minority clusters. In addition, we also provide the results regarding various minimum retention rates for the case when the number of clusters is 10 in Supplementary Fig. S10.

### 3.2 Evaluation of scGPCL on real scRNA-seq datasets

To verify the effectiveness of scGPCL on real-world applications, we conduct experiments on real scRNA-seq datasets over various sequencing platforms. Detailed descriptions regarding the datasets are summarized in Supplementary Table S1. Specifically, we utilize nine datasets with various cell counts (i.e. from 777 to 27 499), and verify whether scGPCL is scalable to large datasets.

**Figure 3.**
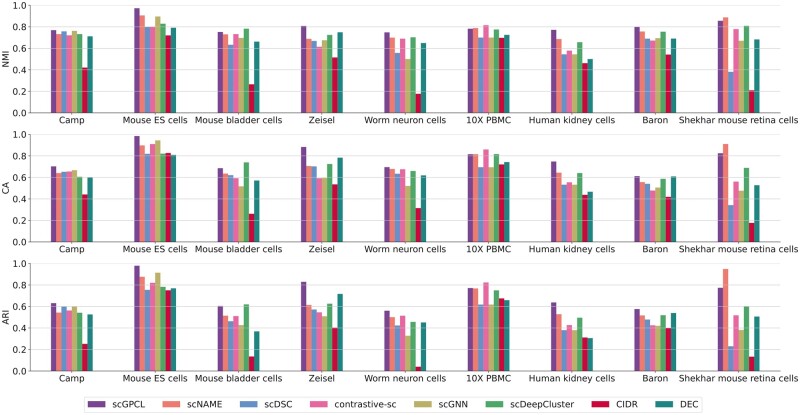
Performance comparisons of scGPCL and other baselines on the nine real scRNA-seq datasets.

Figure 3 shows the overall clustering performance on all nine real-world datasets. Through these experiments, we have the following observations:

(i) scGPCL consistently outperforms the state-of-the-art baselines on six datasets (i.e. Camp, Mouse ES cells, Zeisel, Worn neuron cells, Human kidney cells, and Baron), and achieves competitive scores on the three remaining datasets (i.e. Mouse bladders cells, 10X PBMC, and Shekhar mouse retina cells) compared with scDeepCluster, contrastive-sc, and scNAME, respectively. In addition, we conduct Wilcoxon signed ranks test in Supplementary Table S4 to show that the performance of scGPCL significantly outperforms that of baselines over all the nine datasets and also provide paired t-test results in Supplementary Table S5. (ii) It is worth noting that scGPCL outperforms contrastive-sc that only leverages instance-wise contrastive learning with a naive augmentation strategy that simply masks some gene expression values. We argue that more advanced augmentation strategy is required to fully leverage the benefit of contrastive learning, and that using the relational information between cells is beneficial. (iii) Thanks to the auxiliary mask estimation process that overcomes the limitation of augmentation strategy of contrastive-sc and neighborhood contrastive loss by achieving compact clusters, scNAME generally outperforms the contrastive-sc. However, it shows lower performance than that of scGPCL. We argue that this is due to the fact that scNAME overlooks any relational information between cells inherent in the scRNA-seq data. (iv) scDSC that enhances scDeepCluster using GNNs with a cell–cell graph performs worse than scDeepCluster, which implies that simply infusing relational information through a cell–cell graph cannot generally achieve positive effects in many cases. However, scGPCL generally outperforms scDSC by introducing a bipartite cell–gene graph on which a two-layer GNN encoder is applied to reflect the relational information between cells through the message passing scheme. Additionally, we also provide sensitivity analyses by varying the number of clusters on these datasets in Supplementary Fig. S11 demonstrating that scGPCL generally shows the robust performance when the number of clusters from model is not matched with the true cluster numbers.

To qualitatively compare scGPCL and the baseline methods, we also visualize the cell representations obtained from each method using t-SNE in Supplementary Fig. S12, where each point represents the representation of a cell and the color denotes the cell types to which the cell belongs. The visualization results show that scGPCL tends to well separate the cell types across nine real scRNA-seq datasets. Note that even if these results may not be appropriate to directly compare among the methods due to inevitable information loss while converting a high dimensional space to a low dimensional space, we argue that it is sufficient to demonstrate that scGPCL learns cell representation that preserves the cell type information accurately.

### 3.3 Model analysis

**Figure 4.**
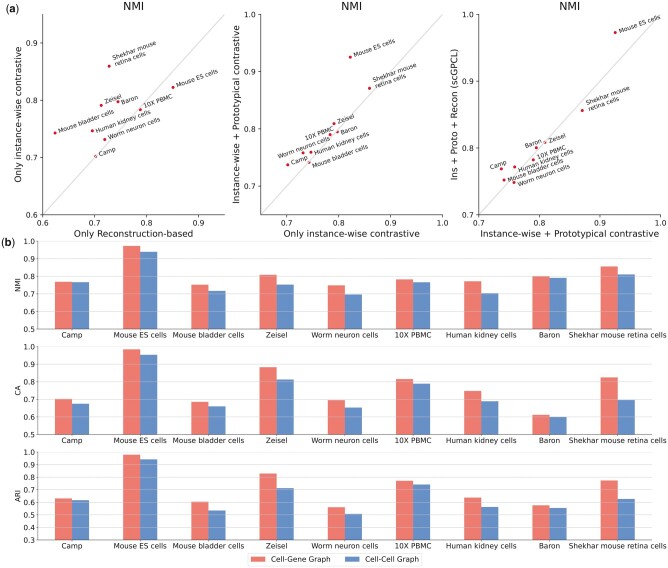
(a) Ablation studies regarding each component in scGPCL and (b) the types of the underlying graph (i.e. cell–gene graph versus cell–cell graph) for scGPCL.

We conduct ablation studies to clarify the benefits of each component of scGPCL. In Fig. 4 and Supplementary Fig. S13, we test each loss function in the pre-training phase and have the following observations: (i) Contrastive learning scheme consistently shows an increased performance compared with the one that only uses the reconstruction-based loss (i.e. Recon only) except for 10X PBMC and Worm neuron cells datasets. (ii) Using prototypical contrastive loss (i.e. Ins+Proto) is more beneficial than using only instance-wise contrastive learning (i.e. Ins only) because it can alleviate the sampling bias and help to infuse cell type information during the pre-training phase. Additionally, Supplementary Fig. S14 also verifies the effect of prototypical contrastive loss by showing that using prototypical contrastive loss (i.e. scGPCL) is generally more beneficial than not using it. (iii) Adding the reconstruction loss (i.e. scGPCL) is beneficial in some cases, however, it does not show consistent performance improvements. Through these results, we argue that the reconstruction loss can be considered as an auxiliary loss that is helpful in stabilizing the performance, but not the main component of scGPCL. Further analyses can be found on the following Supplementary Figures and Notes: Supplementary Fig. S15 and Supplementary Notes S1.3.2 for analysis regarding different reconstruction losses, Supplementary Fig. S16 for benefits of fine-tuning phase, Supplementary Figs S17–S19 for sensitivity analysis of balance coefficients (i.e. *λ*_1_, *λ*_2_, and *λ*_3_) Supplementary Fig. S20 and Supplementary Notes S1.3.3 for ablation study to augmentation strategies discussion, and Supplementary Figs S21–S24 and Supplementary Notes S1.3.4 for discussion regarding model hyper-parameters.

Furthermore, we conduct ablation studies on the types of the underlying graphs (i.e. cell–gene graph versus cell–cell graph). Our goal is to verify our claim that it is better to leverage a cell–gene graph to maintain the quality of the constructed graph compared with a cell–cell graph which may have an adverse effect when the quality of constructed graph dropped due to the instability of the pre-computed cell–cell similarity. To this end, in Fig. 4b, we conduct experiments by changing the type of the underlying input graph of scGPCL to the cell–cell graph, which is constructed based on 10 nearest neighbors of each cell based on the Pearson correlation as the similarity measure following scGNN and scDSC, and compare its performance with that of the original scGPCL that uses a cell–gene graph. More precisely, both of them have same decoder structures, but encode the cell representations using GNNs on cell–cell graph and GNNs on cell–gene graph, respectively. We observe that scGPCL with a cell–gene graph as the input consistently outperforms that with a cell–cell graph, which demonstrates that the cell–gene graph better helps to infuse the inherent relational information between cells. In addition, we also check the performance of scGPCL on cell–cell graph by varying the number of neighbors on cell–cell graph on Supplementary Fig. S25. It shows that the performance of scGPCL on blackcell–cell graph is sensitive to the number of neighbors that is hard to find proper values in practice and performance of scGPCL on cell–gene graph generally outperforms that of cell–cell graph demonstrating that encoding cell representations using cell–gene graph is better choice in our framework.

Moreover, we verify the scalability and robustness with respect to the number of cells by conducting experiments over various numbers of cells. These experimental results can be found in Supplementary Figs S26–S28 with Supplementary Notes S1.4. In addition, we also perform three downstream tasks, namely marker gene identification, gene set enrichment analysis, and cell–cell communication analysis, to provide the biological interpretability of the clustering results and report them on the Supplementary Figs S29–S33 with Supplementary Note S1.5.

## 4 Conclusion

In this article, we propose a graph-based prototypical contrastive learning method aiming at clustering cells in the scRNA-seq data that fully leverages the relational information between cells. Instead of relying on the feature information of each cell (i.e. gene expression value), scGPCL learns the cell representations using GNNs applied on a bipartite cell–gene graph to reflect the natural relationship between cells inherent in the scRNA-seq data. Moreover, scGPCL adopts instance-wise contrastive learning scheme to fully leverage the relational information as well as prototypical contrastive loss to alleviate the limitation of instance-wise contrastive loss (i.e. sampling bias). Through extensive experiments on both simulated and real scRNA-seq datasets, we demonstrate the effectiveness and robustness of scGPCL under common real-world challenging scenarios: (i) gene expression matrix is highly sparse, (ii) gene expression values have low signal strength required for clustering, and (iii) the size of cell clusters is imbalanced. Moreover, we show the scalability of scGPCL on large datasets, which demonstrates the practicality of scGPCL in reality.

## Supplementary Material

btad342_Supplementary_Data

## Data Availability

The data underlying this article are available in https://github.com/Junseok0207/scGPCL/.
