# Supplementary material for: Deep single-cell RNA-seq data clustering with graph prototypical contrastive learning
Source: Bioinformatics. 2023 May 26;39(6):btad342. doi: 10.1093/bioinformatics/btad342 (PMC10246584; doi:10.1093/bioinformatics/btad342)
Supplement: btad342_Supplementary_Data [file btad342_supplementary_data.pdf]

Supplementary Materials - Deep single-cell RNA-seq data clustering with graph prototypical contrastive learning

Junseok Lee[a], Sungwon Kim[b], Dongmin Hyun[c], Namkyeong Lee[a], Yejin Kim[d], Chanyoung Park[a,e,*]

[a] *Dept. of Industrial and Systems Engineering, KAIST, Daejeon, 34141, Republic of Korea*
[b] *Graduate School of Data Science, KAIST, Daejeon, 34141, Republic of Korea*
[c] *Institute of Artificial Intelligence, POSTECH, Pohang, 37673, Republic of Korea*
[d] *Center for Safe Artificial Intelligence for Healthcare, School of Biomedical Informatics, University of Texas Health Science Center at Houston, Houston, Texas, 77030, USA*
[e] *Graduate School of Artificial Intelligence, KAIST, Daejeon, Republic of Korea*

1. Supplementary Notes

1.1. Implementation Detail.

scGPCL is implemented in Python 3 (version 3.8.8) using PyTorch (`https://pytorch.org/`) with Pytorch Geometric (`https://github.com/pyg-team/pytorch_geometric`) packages. For all experiments, except for the learning rate that is searched in {1e-2, 1e-3, 1e-4, 1e-5}, all the hyper-parameters are fixed to default values and shared across all the datasets. Specifically, we use a two-layer GraphSAGE [12] encoder and two-layer MLP decoder where the hidden dimensions are (256, 64), and (64, 256), respectively. To train our model, we use adamW [18] optimizer. The batch size (i.e., the number of anchor cell nodes) is set to 1024 and the number of cell nodes and gene nodes on the subgraph are set to 2048 and 1024, respectively, where these nodes are sampled using HGSampler in Pytorch Geometric. Through all experiments, the maximum pre-training epoch and fine-tuning epoch are fixed to 200 and 100, respectively, and the threshold for convergence test for pre-training phase (i.e., r) and fine-tuning phase (i.e. tol) are set to 0.99 and 0.1, respectively. The temperature hyperparameter for contrastive loss (i.e., τ) is set to 0.25 and the degree of freedom of the Student's t-distribution (i.e., α) is set to 1. For the prototypical contrastive learning, we set the cluster size to K, $1.5K$, and $2K$ where K is ground-truth cluster number in each dataset. In addition, the balance coefficients λ_1, λ_2, and λ_3 are set to 1.0, 0.05, and 1.0, respectively and

*Corresponding author
Email addresses: `junseoklee@kaist.ac.kr` (Junseok Lee), `swkim@kaist.ac.kr` (Sungwon Kim), `dm.hyun@postech.ac.kr` (Dongmin Hyun), `namkyeong96@kaist.ac.kr` (Namkyeong Lee), `yejin.kim@uth.tmc.edu` (Yejin Kim), `cy.park@kaist.ac.kr` (Chanyoung Park)

λ_2 is set to 0 during the first 100 warm-up epochs. All the experiments are conducted on Intel Xeon Silver 4210R CPU and NVIDIA GeForce RTX 3090 (24GB).

1.2. Evaluation metrics

To compare the clustering performance of the scGPCL with the state-of-the-art baselines, we use four standard evaluation metrics, i.e., NMI, CA, ARI, and F1-score, defined as follows:

1) Normalized Mutual Information (NMI) evaluates the clustering quality by measuring the uncertainty of predicted class labels. Specifically, when ground-truth cell type S and the cluster assignment of models C are given, NMI is calculated as follows:

$$NMI = \frac{2 \times I(S;C)}{[H(S) + H(C)]} \tag{1}$$

where $I(\cdot,\cdot)$ denotes the mutual information between two distributions, and H denotes the entropy function. NMI is ranged 0.0 and 1.0 and becomes higher when the predicted cluster assignment and ground-truth are well aligned.

2) Clustering Accuracy (CA) measures the clustering performance in a manner similar to that of the supervised classification. More precisely, we find the best matching function which maps the predicted cluster assignments to the ground-truth cell types, and then calculate the alignment between them. CA is computed as follows:

$$CA = \max_{m} \frac{\sum_{i=1}^{N} \mathbb{1}_{[s_i = m(c_i)]}}{N} \tag{2}$$

where N is the number of instances and m is a matching function which maps the predicted cluster assignments to the ground-truth cell types, and s_i and c_i denote the ground-truth cell type and predicted cluster assignment of the i-th cell, respectively.

3) Adjusted Rand Index (ARI) adjusts the Rand Index (RI) which is defined as

$$RI = \frac{a+b}{{}_{N}C_2} \tag{3}$$

where a represents the number of pairs that successfully belong to the same cluster, while b represents the number of pairs correctly labeled as not belonging to the same cluster. ARI is computed as follows:

$$ARI = \frac{RI - E[RI]}{max(RI) - E[RI]} \tag{4}$$

where $E[RI]$ means the expectation of RI. ARI is ranged between -1 and 1, and becomes larger when the agreement between ground-truth cell types and predicted cluster assignment is similar.

4) F1-score is the harmonic mean of precision and recall which is suitable for the imbalanced data where the precision is the proportion of positive instances out of positively predicted instances and recall is the proportion of positively

predicted instances out of all positive instances on the binary classification setting. F1-score is computed as follows:

$$F1\text{-}score = \frac{2(Precision \times Recall)}{Precision + Recall} \tag{5}$$

To measure the clustering performance, we also find the best matching function to map the predicted cluster assignments to the ground truth cell types and generalize to the multi-class setting by applying micro and macro average scheme (i.e., Micro-F1 and Macro-F1, respectively).

1.3. Additional Model Analysis

1.3.1. Comparison between different graph structure

To validate the impact of the input data types for the cell clustering task, in Supplementary Figure S1, we compare the clustering performance of encoders applied on three different input data types (i.e., Multi-layer Perceptron (MLP) on cell features, GNNs on a cell-cell graph, and GNNs on a cell-gene graph). We observe that GNNs on both the cell-cell graph and the cell-gene graph show good performance over relatively low dropout rates (i.e., $< 40\%$) thanks to the reflection of the relational information between cells, while the performance of GNNs on the cell-cell graph significantly degrades when the dropout rate is higher than 40%. We argue that this is because the highly sparse gene expression matrix makes it hard to accurately compute the similarities between cells, which eventually leads to the drop in the quality of the cell-cell graph. In consequence, this incurs a negative effect on the neighborhood aggregation scheme of GNNs, which eventually results in a poor performance of GNNs on the cell-cell graph.

1.3.2. Ablation study regarding different reconstruction losses

We compare the three reconstruction-based losses (i.e., negative ZINB likelihood loss, NB loss and MSE loss). In Supplementary Figure S15, we observe that when they are applied to scGPCL, all of them had similar clustering performance, in contrast to the case when they are applied to existing methods (i.e., DCA, scDeepCluster, and scDSC) as shown in [9, 22, 11] We attribute this to the fact that, unlike existing methods, scGPCL learns the cell representations using both the instance-wise and the prototypical contrastive losses, and thus the reconstruction loss is not the main component of scGPCL in terms of learning the cell representation. Through these results, we verify that using the MSE loss can be an option to learn cell representations, which enjoys a faster training time than ZINB loss as reported in Supplementary Figure S27. In addition, we show the benefits of the fine-tuning phase of scGPCL and provide the sensitivity of the associated balance coefficient (i.e., λ_3) in Supplementary Figure S16 and S19, respectively.

1.3.3. Ablation study regarding different augmentation strategies

Recall that the augmentation scheme is composed of two steps: sampling two different subgraphs and feature masking. Hence, to verify the effectiveness

of the augmentation scheme, we conduct ablation studies on each step in Supplementary Figure S20. First, to verify the effectiveness of the feature masking step, we run scGPCL without masking features of the bipartite cell-gene graph (we refer to this as **w/o feature masking**). Second, to verify the effectiveness of sampling different subgraphs, we sample a single subgraph and use it on both views (we refer to this as **same subgraph**). Experiment results show that removing either one of the two steps generally degrades the performance, and especially sampling the same subgraph on both views incur a large performance drop. Through theses results, we argue that both feature masking and subgraph sampling steps are important, and especially subgraph sampling is a key augmentation strategy in scGPCL. It is worth noting that the subgraph sampling step not only plays a role as an augmentation strategy, but also alleviates the memory issue incurred by large bipartite cell-gene graph.

1.3.4. Hyper-parameter sensitivity analysis

We provide several sensitivity analyses results to check the effect of each hyperparameter in scGPCL. First of all, we report the performance of scGPCL with a different number of anchor cell nodes (i.e., batch size) in Supplementary Figure S21. It does not show a significant change in performance as the number of anchor cell nodes is changed, which shows that scGPCL is robust to the number of anchor cell nodes.

In Supplementary Figure S22, we check whether the number of clustering processes (i.e., T) on prototpyical contrastive loss affects the performance of scGPCL. We observe that there is no significant change when the number of clustering processes is changed, but there is a trend that conducting clustering many times helps to increase model performance. We argue that it verifies that finding semantically similar groups across various granularities can help the training process of scGPCL. In addition, in Supplementary Figure S23, we also report the sensitivity analysis regarding the cluster sizes on prototypical contrastive loss. It shows that it is better to select the number of clusters similar to the number of ground truth clusters, but it shows robust performance within a reasonable range.

Finally, in Supplementary Figure S24, we generate more views to sample more negative samples from differently augmented graphs. Through this experiment, we check that using more views does not improve the performance of scGPCL. We argue that it is due to the fact that using two views in scGPCL already provides sufficient negative samples for training.

1.4. Scalability of the scGPCL

In practice, the size of single-cell data increases as the sequencing technology advances, and accordingly, the scalability with respect to the number of cells is one of the important evaluation factors when comparing various models. To verify the scalability of scGPCL, we generate a simulated dataset by varying the number of cells from 1K to 100K (i.e., [1K, 5K, 10K, 30K, 50K, 70K, 100K]) with 3000 genes and the other hyper-parameters are set to default values. In

Supplementary Figure S26a, we observe that the running time of scGPCL in the pre-training phase and fine-tuning phase does not increase exponentially as the number of cells increases, and even shows reasonable a running time when the number of cells is extremely large (i.e., total running time of scGPCL for 100K cells is 8,887 seconds). Additionally, we report the performance of scGPCL as the number of cells increases in Supplementary Figure S28 to show that it can obtain accurate clustering performance regardless of the number of cells. Through these results, we verify the scalability of scGPCL from both of the efficacy and efficiency perspectives. This implies that scGPCL can be applied to large-scale scRNA-seq datasets, and thus practical in reality.

1.5. Biological analysis

1.5.1. Marker gene identification

To provide the biological interpretability of the clustering results obtained from scGPCL, we report the marker gene identification result [24] on the Zeisel dataset. Specifically, we find the top 10 differentially expressed genes (DEGs) using the Wilcoxon rank sum test based on the clusters detected by each method and compute the overlap with the gold standard cell types. In Supplementary Figure S29b, DEGs computed based on clustering results obtained from scGPCL highly focus on one cell type except for the 'ependymal' cell type that is hard to detect with the ground truth cell type (upper left). Note that scGPCL succeeds in learning clusters with 'mural' cell type, whereas other baseline methods fail to do so. We argue that it is because, as can be shown in Supplementary Figure S29a, the 'mural' cell type belongs to a minority class with a small number of cells. Through this result, we can annotate cluster 1, 2, 4, 5, 7, 8, and 9 as oligodendrocytes, microglia, interneurons, mural, astrocytes, ca1pyramidal, endothelial, and s1pyramidal cell types, respectively, and once again demonstrate that scGPCL can learn cells that belong to the minority cell type in a biological perspective. We also report the ranking of the DEGs in different clusters in Supplementary Figure S30

1.5.2. Gene set enrichment analysis

To further investigate the biological meaning of the clusters obtained from scGPCL, we perform Gene Set Enrichment Analysis (GSEA) on the mural cluster (i.e., cluster 5) in Zeisel dataset, which is specifically identified by scGPCL compared to the baseline methods. Specifically, we select the top 100 DEGs from cluster 5 annotated as mural cell type and perform KEGG enrichment analysis using these genes. As shown in Supplementary Figure S31, Focal adhesion pathway that plays a crucial role in the development and maintenance of cell-matrix adhesions obtained the highest score. This result is biologically relevant as ephrin-B2 in mural cells plays a role in regulating the formation and turnover of focal adhesions [10]. Additionally, we also report the result of gene ontology (GO) enrichment analysis for biological process, cellular component, and molecular function in Supplementary Figure S32.

1.5.3. Cell-Cell Communication analysis

We conduct cell-cell communication (CCC) inference to validate whether scGPCL accurately captures intercellular relationships. To assess the performance of CCC, we use the Mouse Brain Atlas data [21], which has a gold standard interaction result, following the evaluation protocol suggested by the Open Problems in Single-Cell Analysis group (`https://openproblems.bio/results/cell_cell_communication_source_target/`). Specifically, we employ the liana [7] framework and use CellPhoneDB [8] as the CCC method, using different clustering results from each clustering method. In Supplementary Figure S33, we compare the performance of CCC inference with the odds ratio [2] calculated as the ratio of true and false positives among the top 10% ranked interactions versus the same ratios for the remainder of the interactions. The result demonstrates that scGPCL shows comparable performance as when using the ground truth cell type in detecting interactions between source and target cell types, whereas other baseline methods do not perform well. Based on this finding, we verify that scGPCL effectively captures the cell-cell relationship.

1.6. Baseline methods

The clusterting performance of scGPCL is compared with eight state-of-the-arts baseline methods incorporating graph based methods, instance-wise contrastive learning method, ZINB-based autoencoder methods, graph based deep learning methods, and non-deep learning based methods.

- Clustering through imputation and dimensionality reduction (CIDR) [17] implicitly imputes gene expression data to alleviate the impact of dropout and then calculates dissimilarity matrix. After that, it performs PCoA and clustering using the first few principal coordinates.

- Deep embedded clustering (DEC) [26] jointly optimizes the feature representation and cluster assignments using the self-training strategy.

- Single-cell model-based deep embedded clustering (scDeepCluster) [22] simultaneously learns the cell representation and clustering assignment following DEC, and replaces the MSE loss with negative likelihood of ZINB distribution.

- Single-cell graph neural networks (scGNN) [25] aggregates the relationship between cells in cell-cell graph using GNNs and adopts left-truncated mixture Gaussian (LTMG) model to reflect the heterogeneous gene expression patterns. In addition, it infuses cell-type specific information using cluster autoencoder.

- Contrastive self-supervised clustering of scRNA-seq data (contrastive-sc) [6] adopts instance-wise contrastive learning scheme for the scRNA-seq data by randomly generating two differently augmented views in terms of cell features.

- Structural deep clustering for single-cell RNA-seq data (scDSC) [11] enhances scDeepCluster by jointly optimizing ZINB-based autoencoder and the GNNs on cell-cell graph to aggregate cell-cell relationship.

- scNAME [24] utilizes an auxiliary mask estimation task to grasp the gene pertinence by distinguishing the uncorrupted structure and achieve intra-cluster compacteness and inter-cluster separation using neighborhood contrastive loss that enhances similarity between k-nearest neighbors.

Benchmarking the scDeepCluster [22] and scDCC [23], we differently conduct pre-processing steps on raw read count data following the reported steps on each paper to satisfy their own hypothesis. For all deep learning based baseline methods, we search their best learning rate in {1e-2, 1e-3, 1e-4, 1e-5} and their default learning rate, and the other hyper-parameter setting is set to their default parameter. We utilize the official code for each baseline and it is summarized in Supplementary Table S2.

1.7. Data Availability

- The Camp dataset [3] was sequenced to understand liver bud (LB) by analyzing component lineages. We download the dataset from GSE81252.

- The Mouse Embryonic Stem cells (Mouse ES cells) [16] dataset was obtained from high-throughput droplet-microfludic approach for parallel barcoding, and authors reveal the heterogeneous onset of differentiation after leukemia inhibitory factor (LIF) withdrawl. We obtain the dataset from GSE65525 and we concatenate the data which is seperated by the different days from LIF withdrawal (i.e., 0 days, 2 days, 4 days, and 7 days).

- Mouse bladder cells [13], obtained from Mouse Cell Atlas (MCA) project, was sequenced by Microwell-seq that is a high-throughput scRNA-seq platform. The batch gene removed digital gene expression (DGE) matrix was downloaded from `https://figshare.com/s/865e694ad06d5857db4b` and we define the labels by following the author's annotation of cells.

- The Zeisel dataset [28], which reveals the cell types of the mouse cortex and hippocampus by biclustering method, was counted using unique molecule identifier (UMI) assays. The dataset was obtained from GSE60361 and corresponding cell types are founded on `http://linnarssonlab.org/cortex/`.

- The Worm neuron cells dataset [4], which profiles nearly 50,000 single-cell transcriptomes in the nematode *Caenorhabditis elegans* at the L2 stage, was sequenced by sci-RNA-seq (single-cell combinatorial indexing RNA sequencing) platform. We follow the instruction (`https://atlas.gs.washington.edu/worm-rna/docs/`) to access the raw data and use the subset of neuronal cells. To evaluate alignment with the predicted cluster assignment, we exclude the cells labeled 'Unclassified neurons'.

- The 10X PBMC dataset [29], which profiles the peripheral blood mononuclear cells from a healthy donor, was collected by 10X scRNA-seq platform. We obtained the filtered gene barcode matrix from `https://support.10xgenomics.com/single-cell-gene-expression/datasets/2.1.0/pbmc4k` and use graph based-clustering result as a label following `https://support.10xgenomics.com/single-cell-gene-expression/software/pipelines/latest/output/analysis` to evaluate clustering performance.

- The Human kidney cells dataset [27], which identifies the cellular identity of renal tumors, was profiled by the 10X scRNA-seq platform. We obtained the dataset released by author (`https://github.com/xuebaliang/scziDesk/tree/master/dataset/Young`)

- The Baron dataset [1], which determines individual pancreatic cells from human and mouse, was implemented by a droplet-based method. We obtained the dataset from GSE84133 and conduct experiments using human cells.

- The Shekhar mouse retina cells dataset [20], identifies cell types of the mouse retinal bipolar cells (BCs) was profiled by Drop-seq platform. The dataset was obtained from GSE81904 and corresponding cell types are founded on the `https://scrnaseq-public-datasets.s3.amazonaws.com/index.html?prefix=manual-data/shekhar/`. Then we remove the cells which contain more than 10% mitochondrially derived transcripts and then filter out cells of which ≤ 500 genes are detected. After that we also remove the genes of which ≤ 30 cells are detected or having ≤ 60 transcripts following the preprocessing steps from the Hemberg group (`https://github.com/hemberg-lab/scRNA.seq.datasets/blob/master/R/shekhar.R`).

The statistics of the datasets are summarized in Table S1. Note that except for the Shekhar mouse retina cells dataset which already filters out some cells and genes, we filter out the cells and genes with no count, and keep top 20% highly variable genes following the best practice [19] that selecting highly variable genes between 1000 and 5000 is recommended.

1.8. Future Works

Although scGPCL alleviates the sampling bias of instance-wise contrastive loss using the prototypical contrastive loss, there still remains misaligned cells that may cause inefficiency in the contrastive learning framework due to the unsupervised nature of clustering task. Recently, scDCC [23] extends the task of unsupervised scRNA-seq clustering to a semi-supervised setting by using available prior knowledge. We expect that since existing studies [15, 5, 14] in computer vision domain show that cancelling false negative pairs [5, 14] or adding true positive pairs [15] can help eliminate bias of contrastive learning framework, we expect that scGPCL can be enhanced by guiding unlabeled cells using few labeled cells under the semi-supervised setting.

2. Supplementary Figures

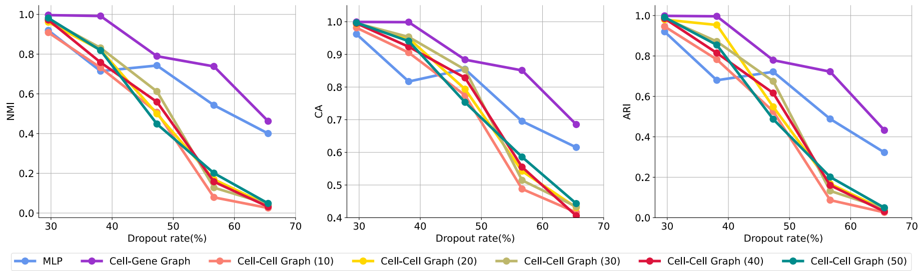

Supplementary Figure S1: Performance comparison of different encoder structures on the simulated dataset over various dropout rates. We construct the autoencoder style models which have the same 2-layer MLP decoders which output the three parameters of ZINB distribution (i.e., mean, dispersion, and dropout probability) and three different encoders that are MLP, GNNs on a cell-cell graph, and GNNs on a cell-gene graph, respectively. More precisely, pearson correlation is used to calculate the similarity between cells to construct cell-cell graphs following scGNN [25] and scDSC [11], and we conduct experiments by varying the number of nearest neighbors (i.e., Cell-Cell Graph (10) represent the 10 nearest neighbors graph based on pearson correlation). The cell-gene graph is constructed by connecting two nodes, if a particular gene is expressed in that cell as proposed on scGPCL.

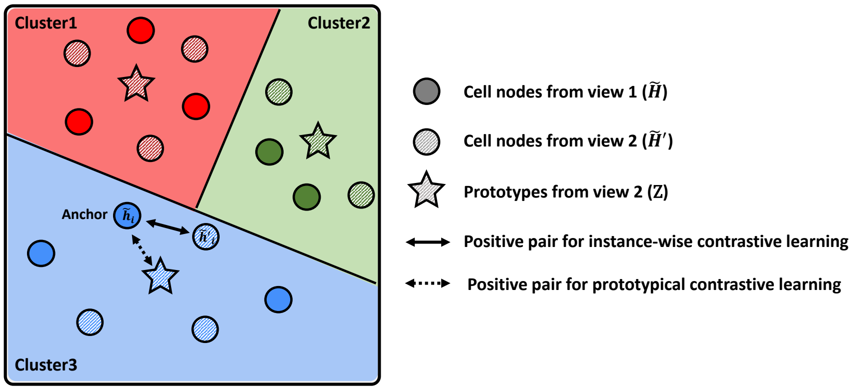

Supplementary Figure S2: An overview of prototypical contrastive learning of cell node i on the representation space. Given an anchor node representation of cell i from view 1 denoted by $\tilde{h}_i$, it pulls another cell node representations of the same cell i from view 2 (i.e., $\tilde{h}'_i$), and pushes all other cell representations from both view 1 and view 2 using contrastive loss to learn a generic representation of each cell. In addition, $\tilde{h}_i$ pulls the prototype assigned for the same cell i from view 2, and also pushes all other prototypes to complement the limitation of contrastive loss which suffers from sampling bias.

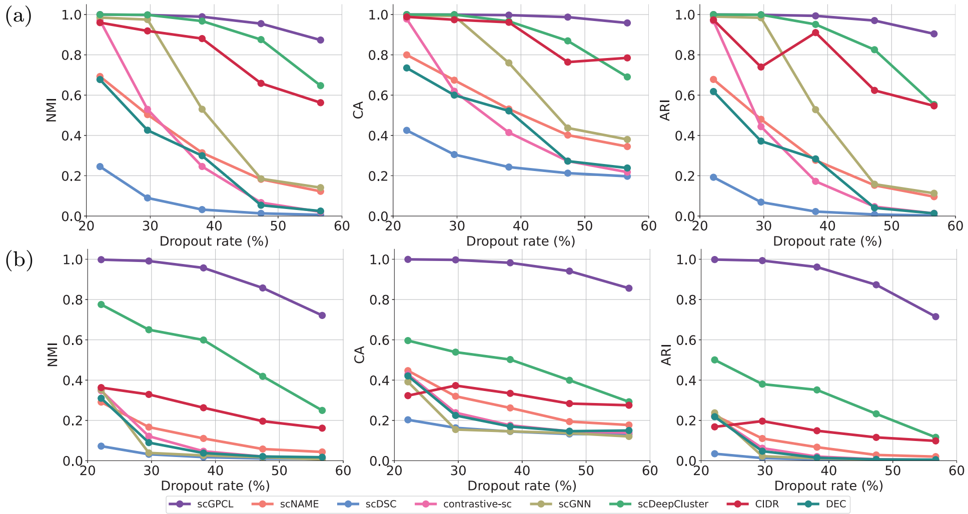

Supplementary Figure S3: Performance comparisons over various dropout rates on simulated data. (a) and (b) show the performances with 6 and 10 clusters, respectively.

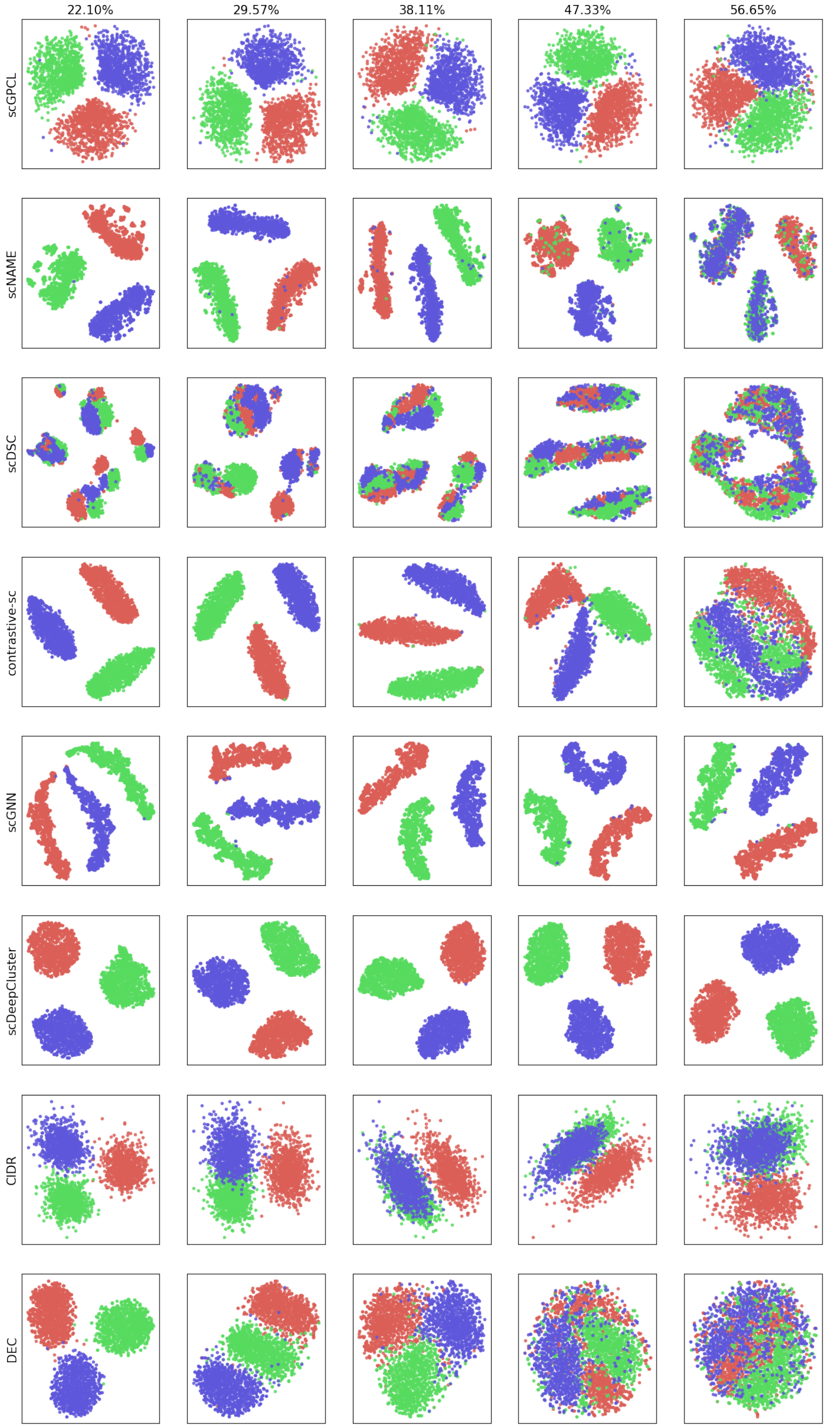

Supplementary Figure S4: Visualization of a simulated dataset with 3 clusters of cells over various dropout rates.

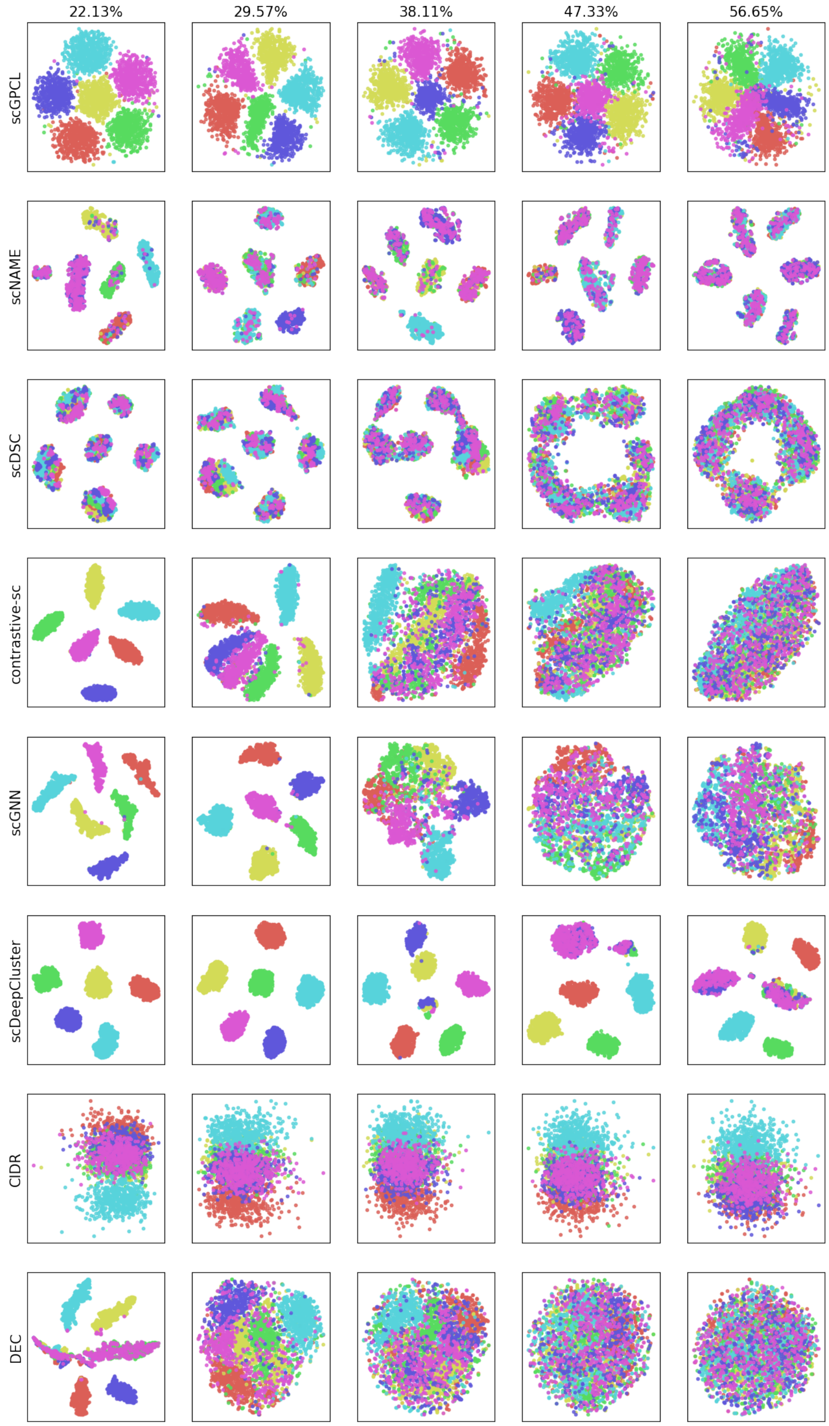

Supplementary Figure S5: Visualization of a simulated dataset with 6 clusters of cells over various dropout rates.

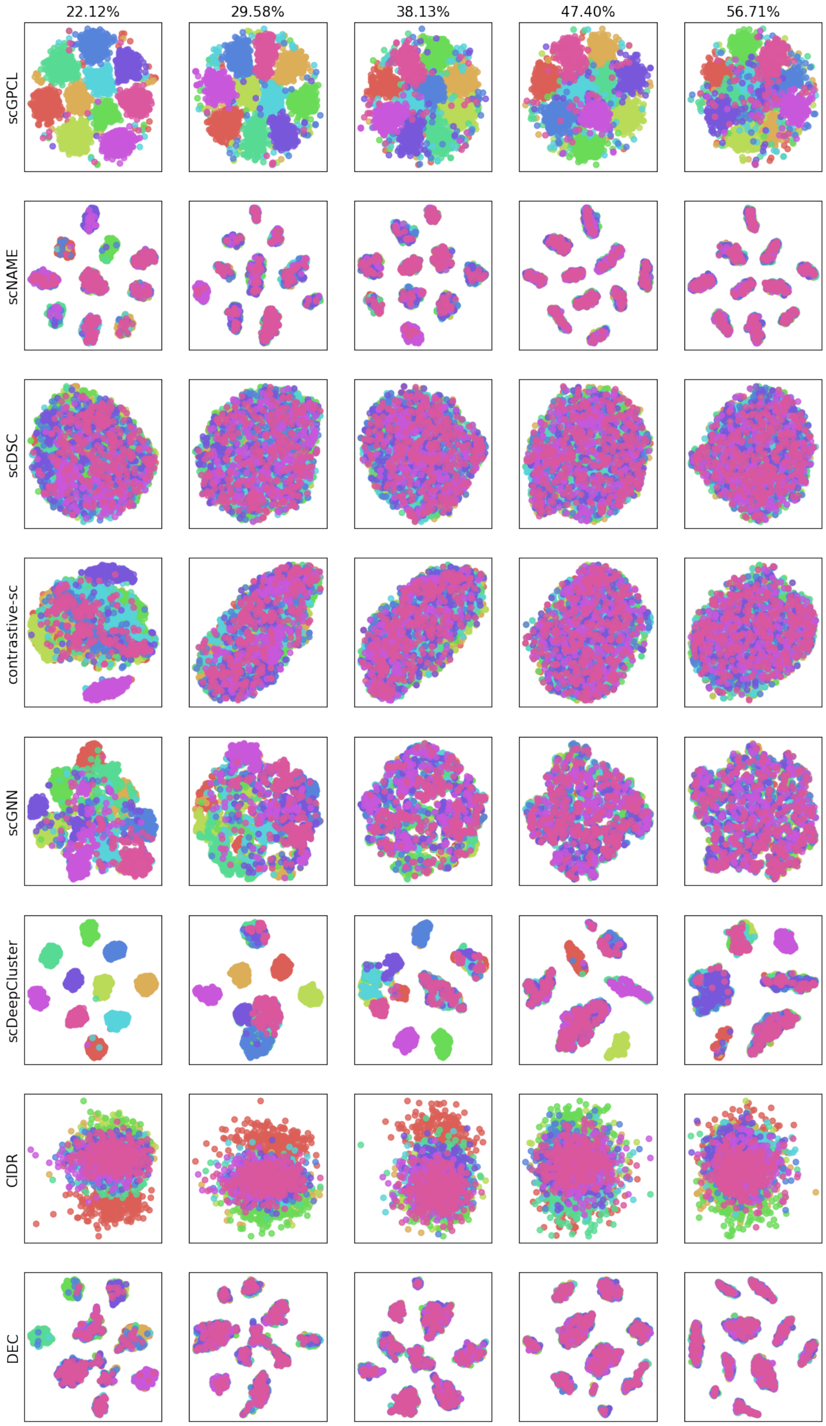

Supplementary Figure S6: Visualization of a simulated dataset with 10 clusters of cells over various dropout rates.

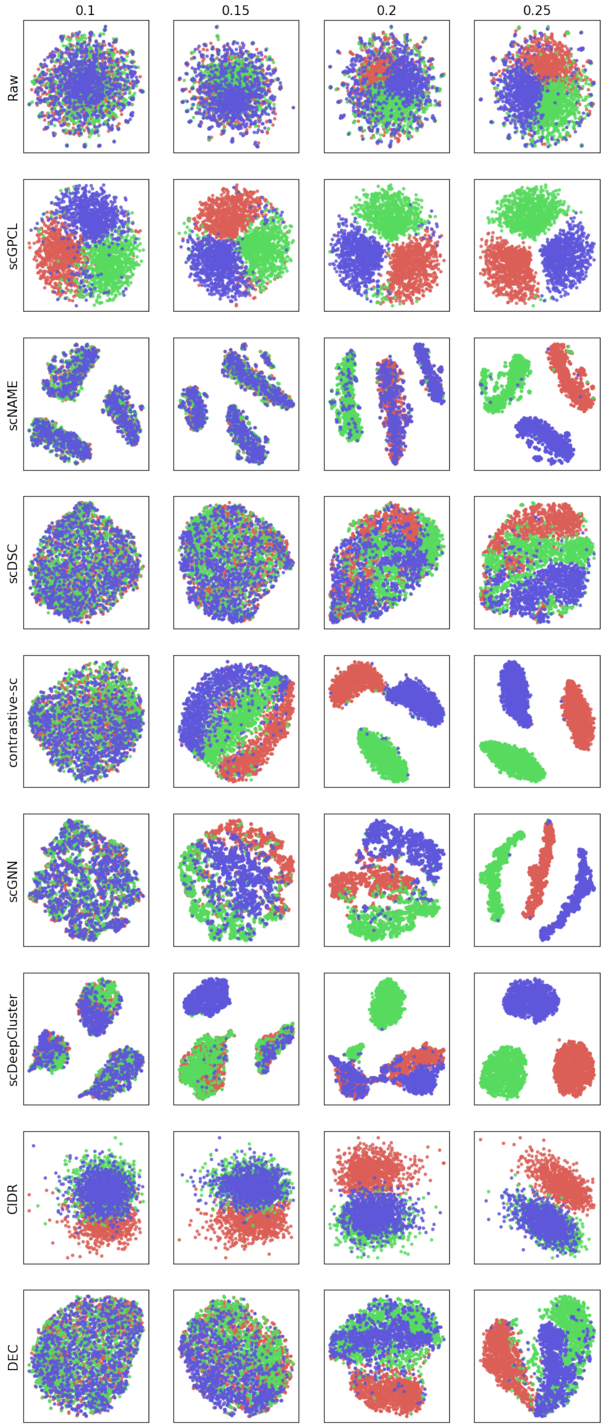

Supplementary Figure S7: Visualization of a simulated dataset with 3 clusters of cells over various sigma (A small sigma indicates low signals for clustering).

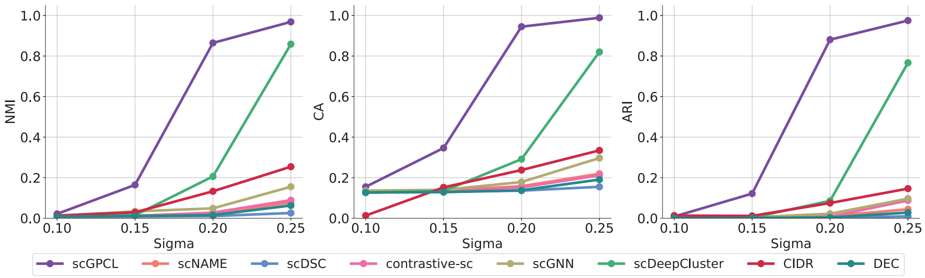

Supplementary Figure S8: Performance comparisons over the various sigmas on the simulated data with 10 clusters of cells (A small sigma indicates low signals for clustering).

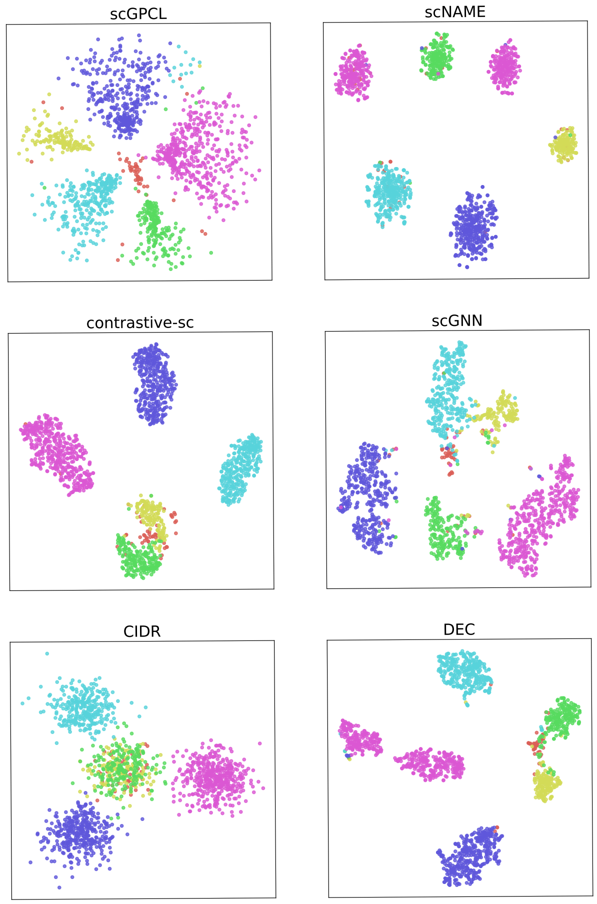

Supplementary Figure S9: Visualization of a simulated dataset in which 1500 cells belong to 6 subgroups when the minimum retention rate is 0.1.

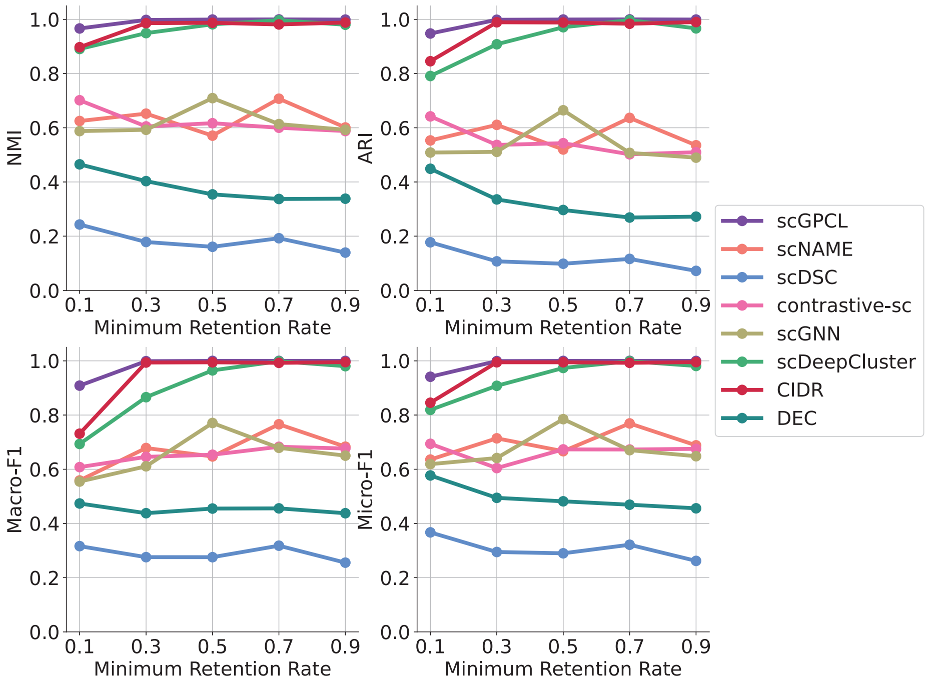

Supplementary Figure S10: Performance comparison over the various minimum retention rates on simulated data with 10 clusters of cells

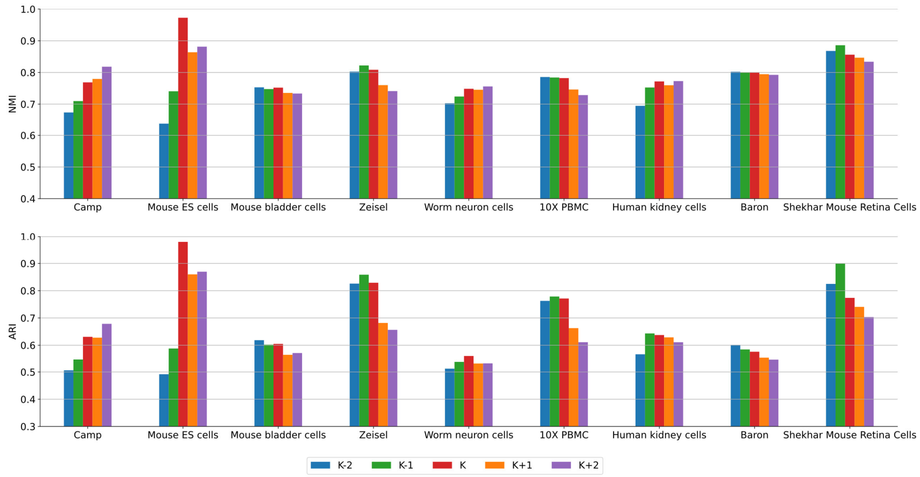

Supplementary Figure S11: Sensitivity analysis regarding the number of clusters. In this experiment, K is the ground-truth cluster number in each dataset, and we report the performance of scGPCL from ten repeated experiments across various numbers of clusters in $\{K-2, K-1, K, K+1, K+2\}$.

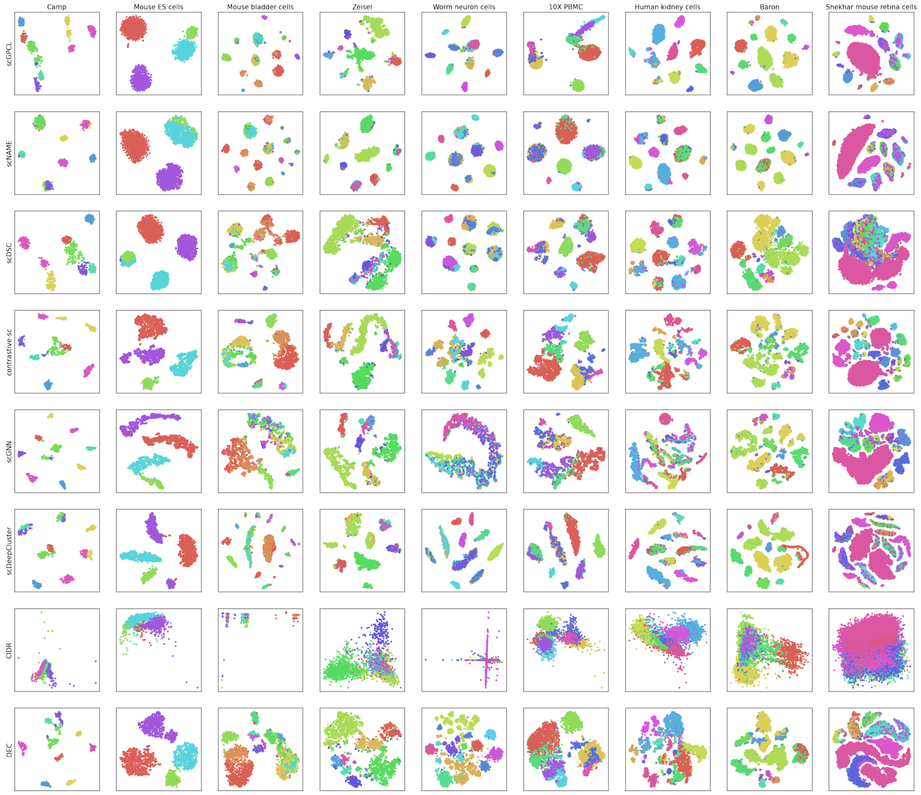

Supplementary Figure S12: Visualization of cell representations obtained from various methods including scGPCL.

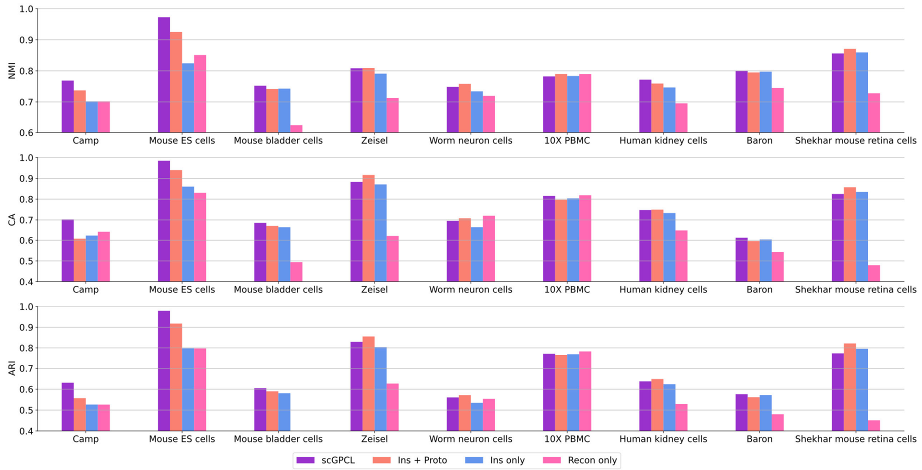

Supplementary Figure S13: Ablation studies of scGPCL during the pre-training phase.

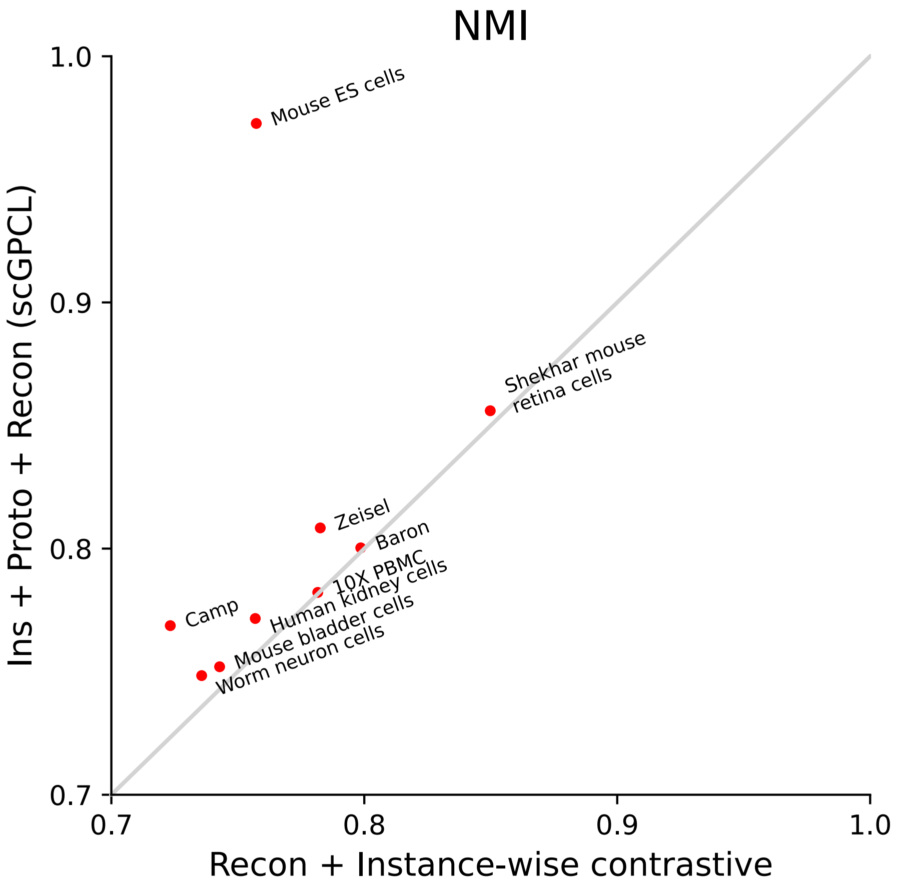

Supplementary Figure S14: Ablation study regarding the prototypical contrastive loss in scGPCL.

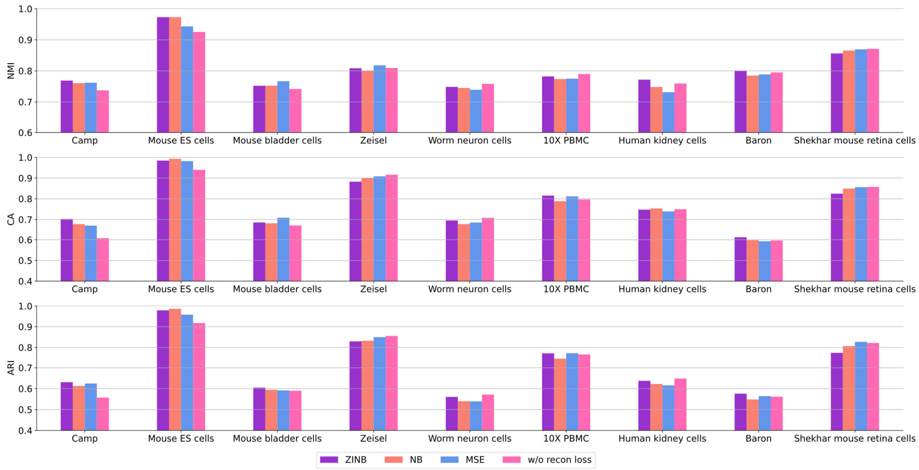

Supplementary Figure S15: Ablation study on scGPCL with different reconstruction-based losses.

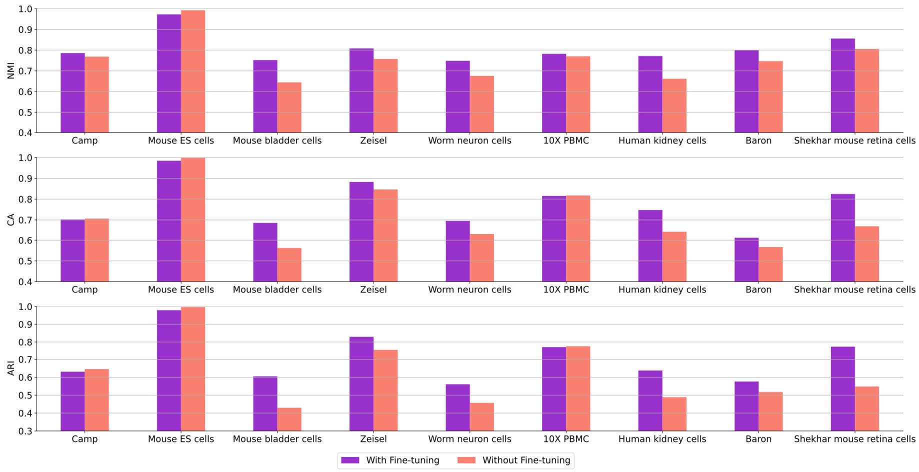

Supplementary Figure S16: Ablation study regarding the benefit of the fine-tuning phase in scGPCL.

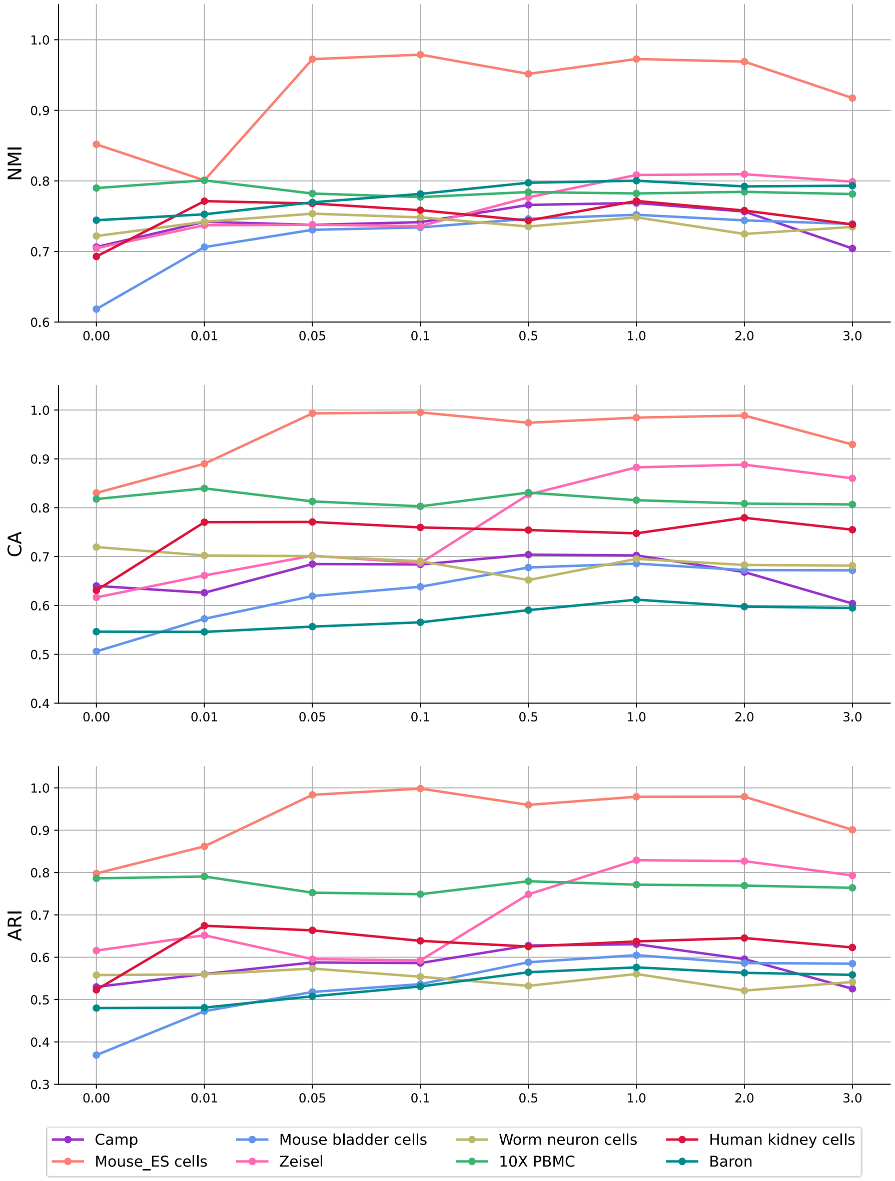

Supplementary Figure S17: Sensitivity analysis regarding the balance coefficient of instance-wise contrastive loss (i.e., λ_1) in scGPCL.

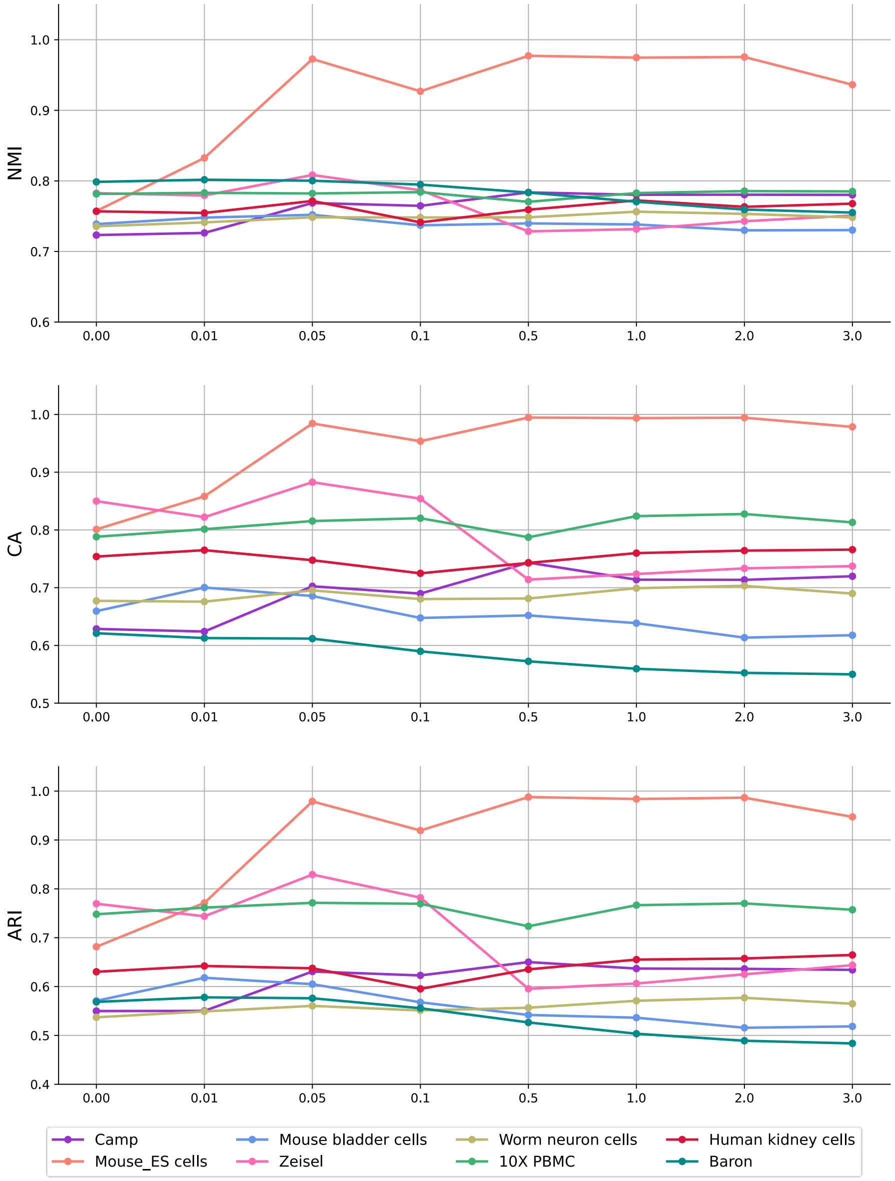

Supplementary Figure S18: Sensitivity analysis regarding the balance coefficient of prototypical contrastive loss (i.e., λ_2) in scGPCL.

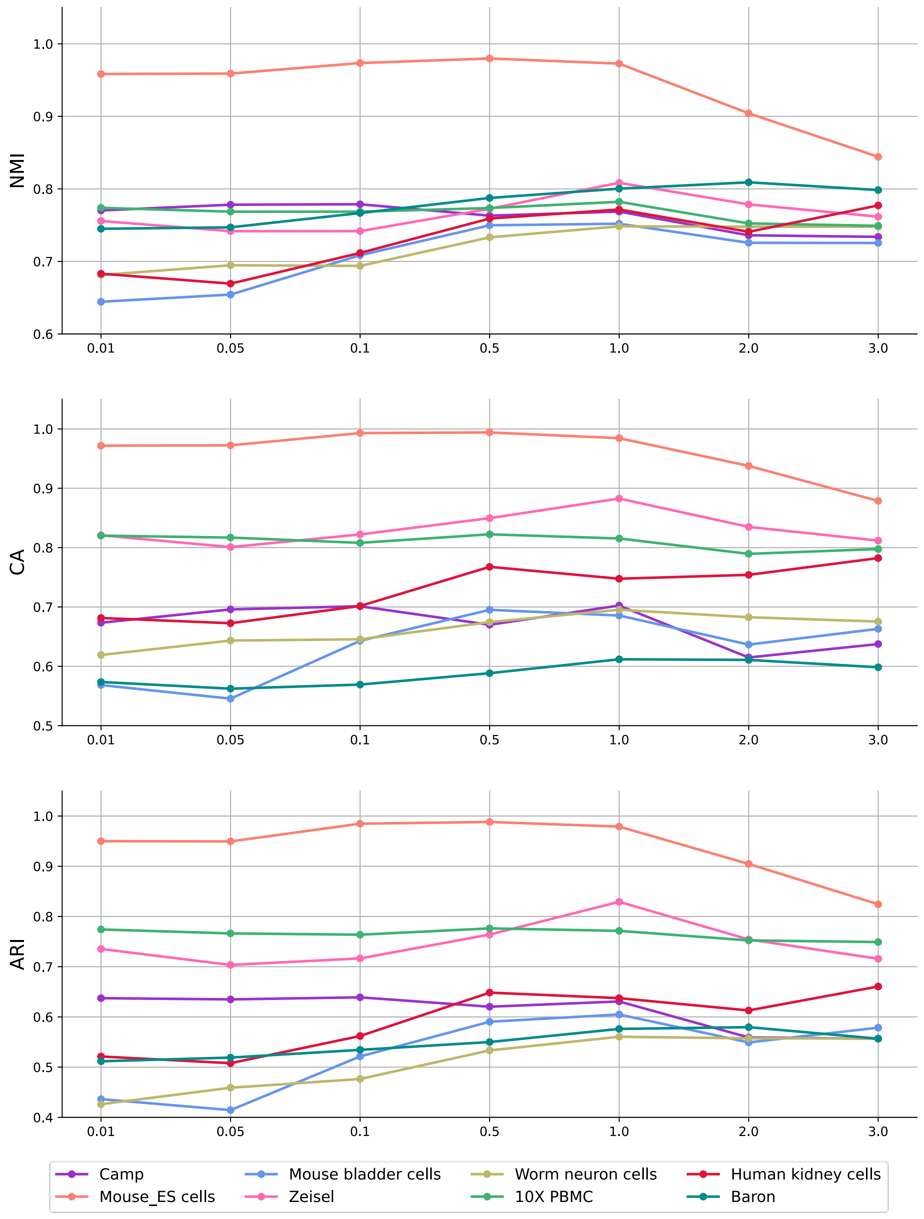

Supplementary Figure S19: Sensitivity analysis regarding the balance coefficient of clustering task-oriented loss (i.e., λ_3) in scGPCL.

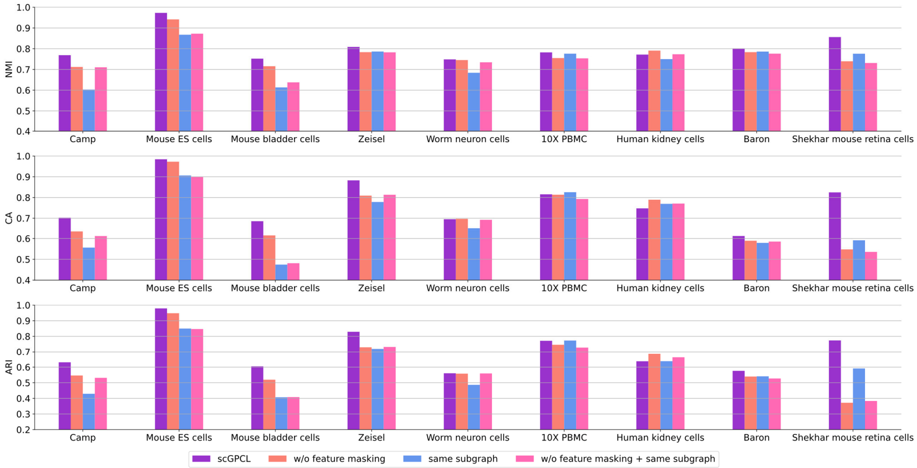

Supplementary Figure S20: Ablation study regarding different augmentation strategies in scGPCL.

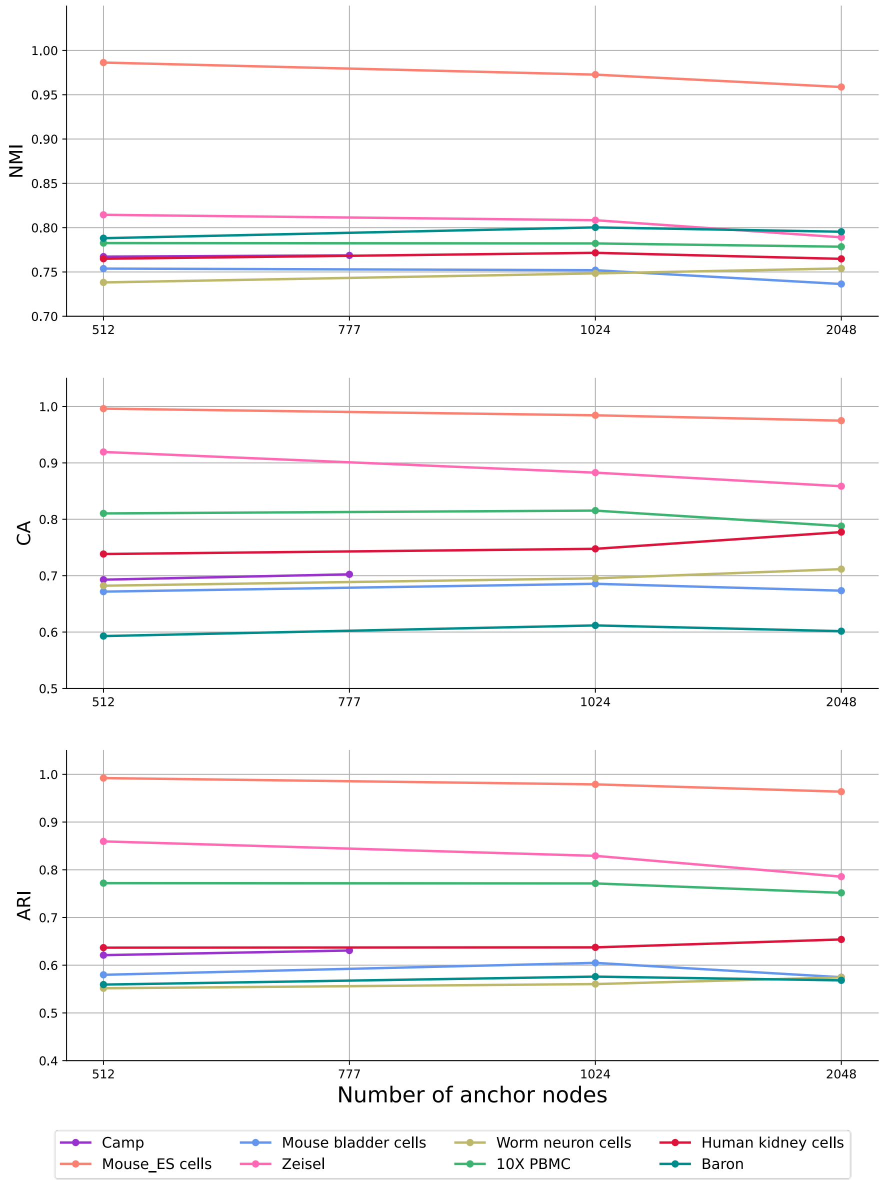

Supplementary Figure S21: Sensitivity analysis regarding the number of anchor nodes (i.e., batch size) in scGPCL. Note that since there exist only 777 cells in Camp dataset, we report the result of 512 and 777 anchor nodes for Camp dataset.

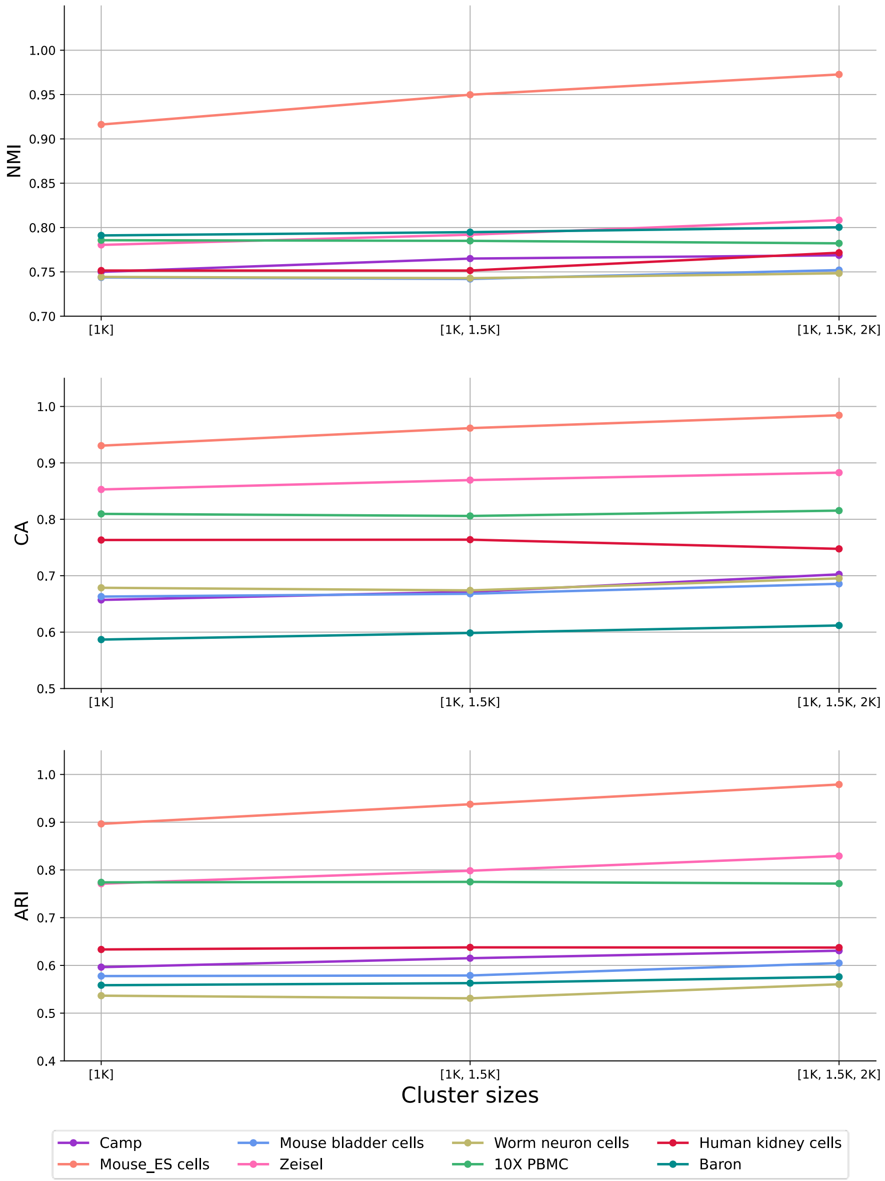

Supplementary Figure S22: Sensitivity analysis regarding the number of clustering processes on prototypical contrastive loss (i.e., T) in scGPCL. In this experiment, K is the ground-truth cluster number in dataset and we report the performance by conducting clustering 1 time with cluster size K (i.e., $[1K]$), 2 times with cluster size K and $1.5K$ (i.e., $[1K, 1.5K]$), and 3 times with cluster size K, $1.5K$, and $2K$ (i.e., $[1K, 1.5K, 2K]$), respectively.

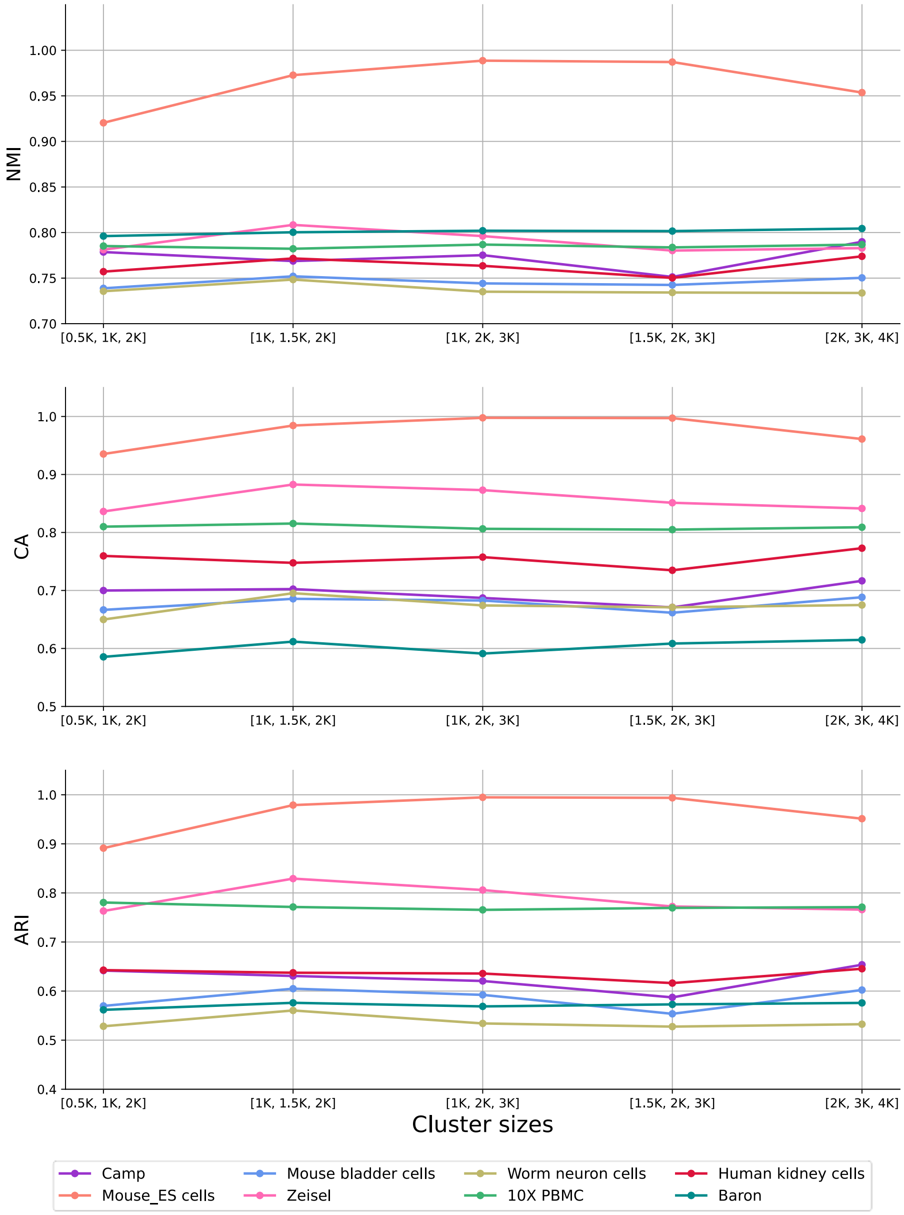

Supplementary Figure S23: Sensitivity analysis regarding the cluster sizes on prototypical contrastive loss in scGPCL. In this experiment, K is the ground-truth cluster number in dataset and $[a,b,c]$ means that we conduct clustering 3 times with cluster size a, b, and c

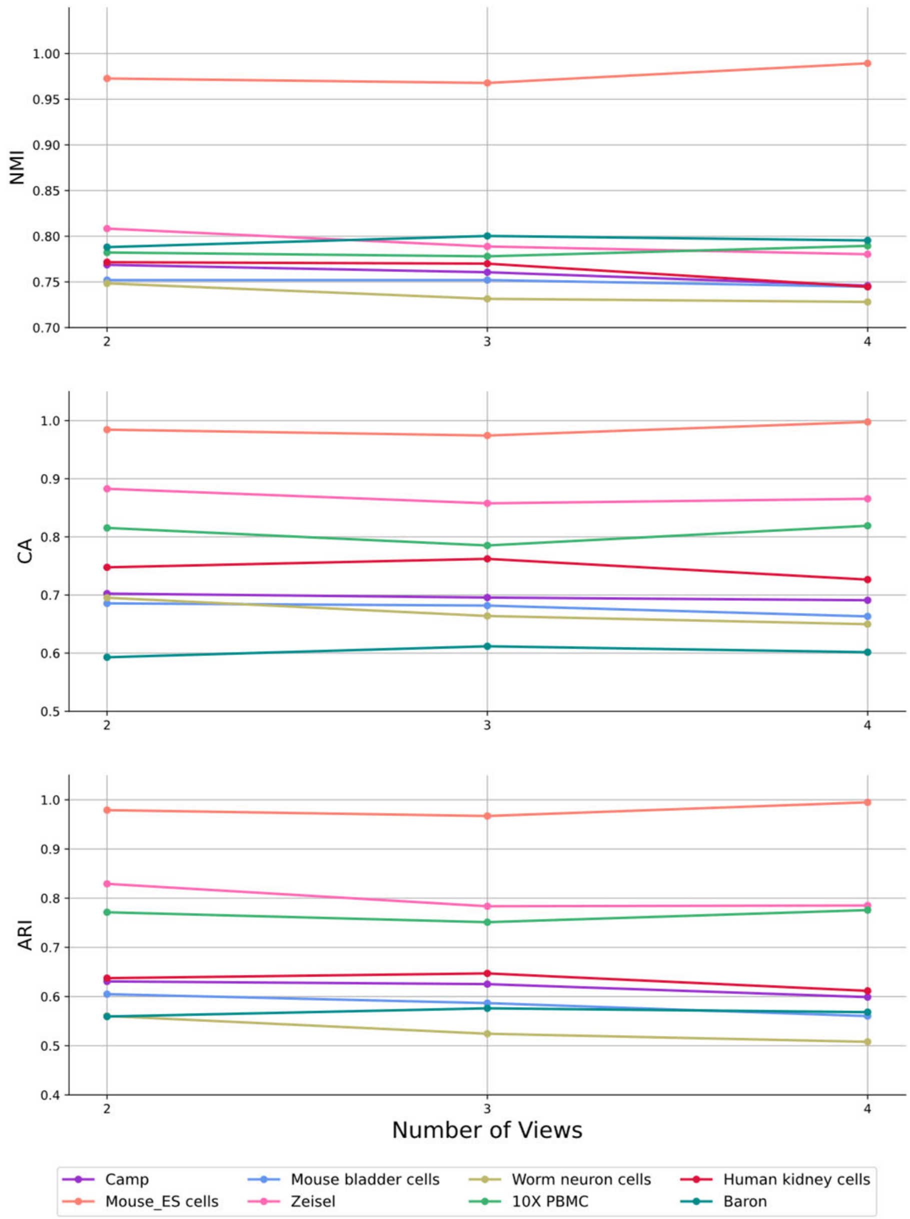

Supplementary Figure S24: Sensitivity analysis regarding the number of views in scGPCL.

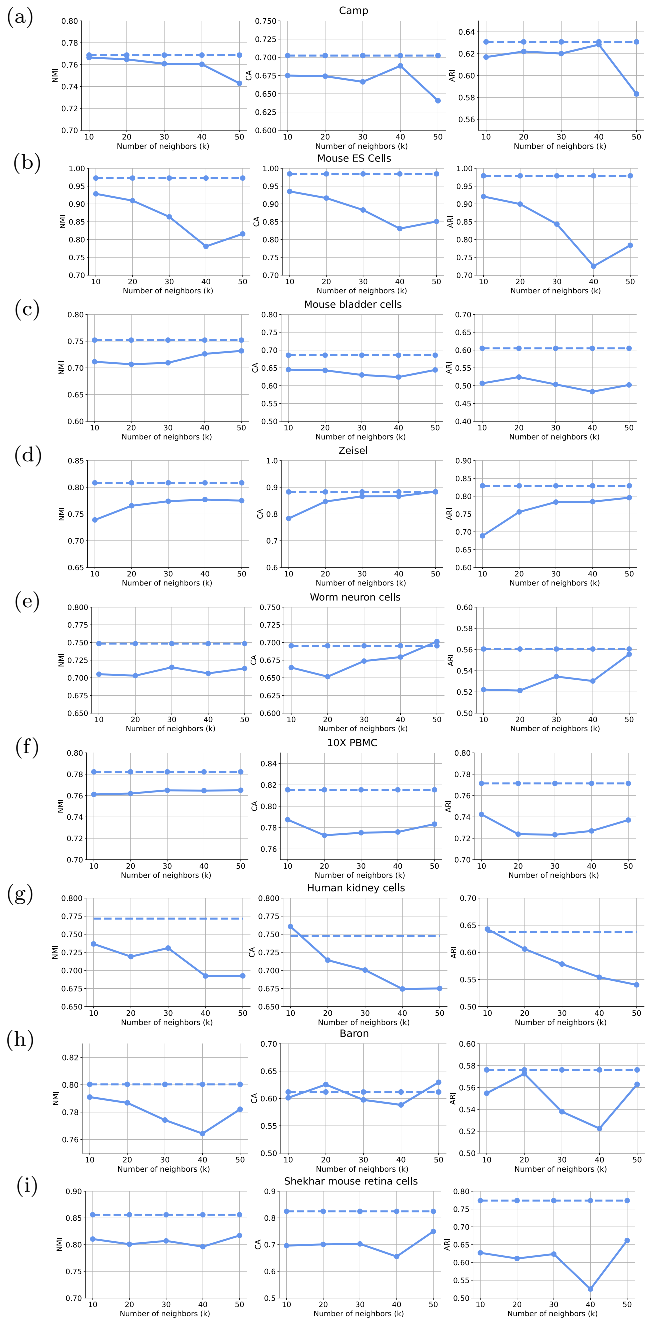

Supplementary Figure S25: Performance of scGPCL using GNN encoder on k-nearest neighbor-based cell-cell graph over various numbers of neighbors (K). Solid lines on (a)-(i) show the performance of scGPCL on cell-cell graph over various numbers of neighbors (k) and dashed lines represent the performance of scGPCL on cell-gene graph.

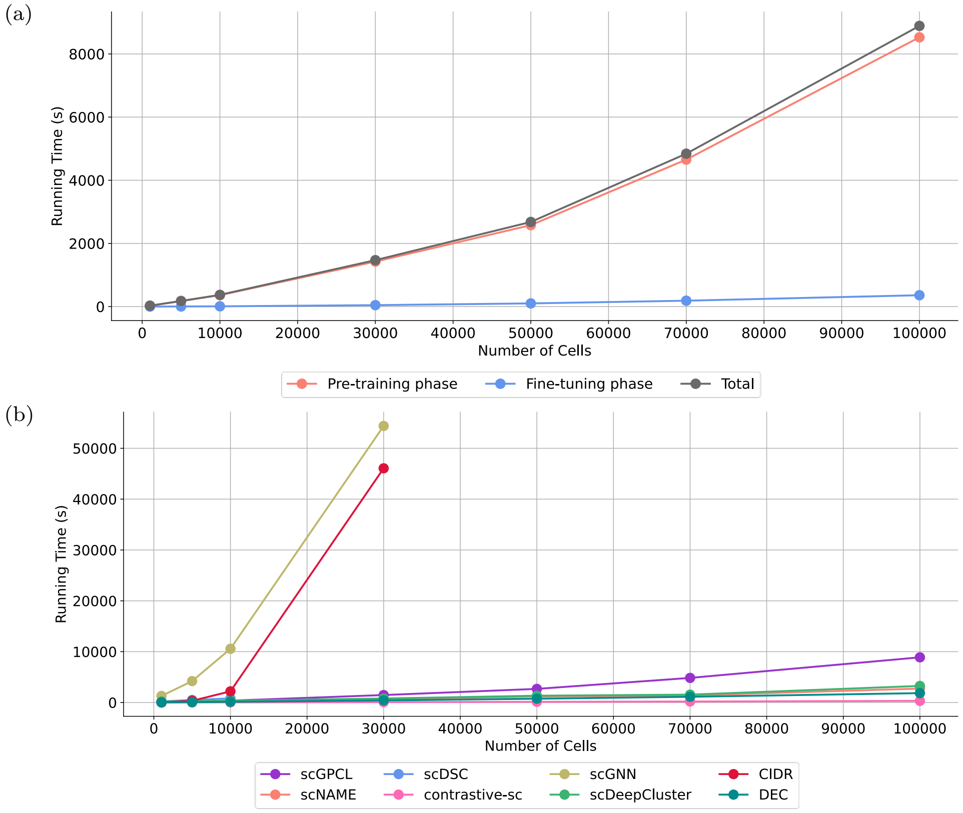

Supplementary Figure S26: (a) The running time of scGPCL over the various number of cells on the simulated data and (b) the running time comparison of scGPCL and other baselines.

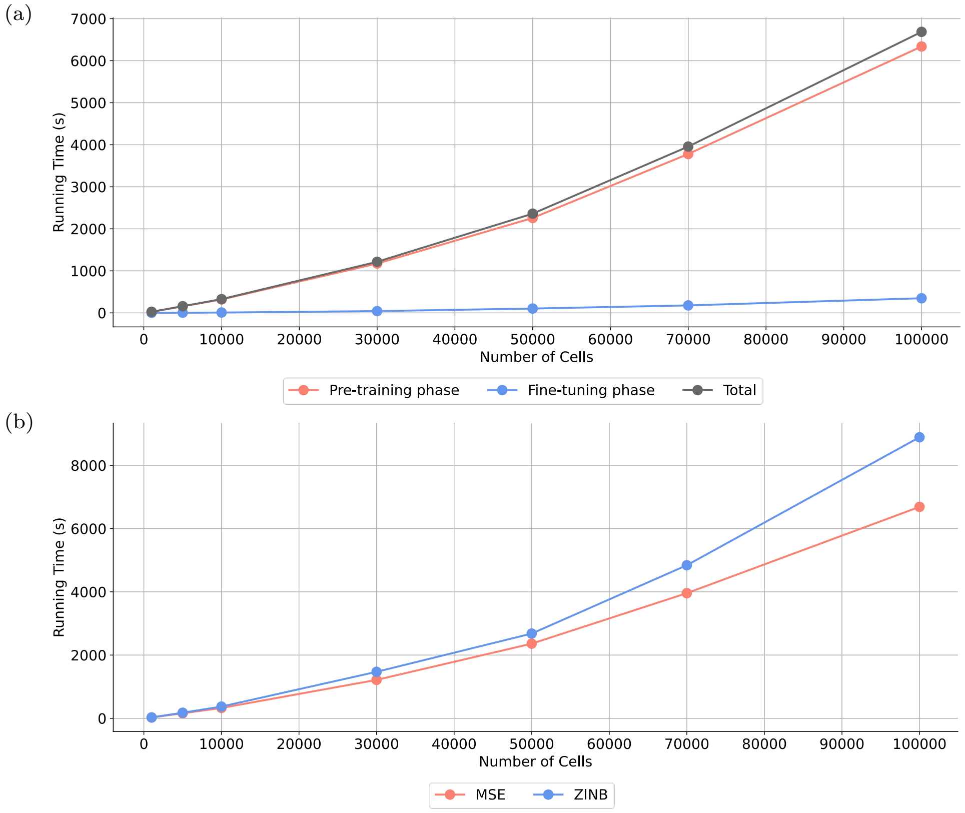

Supplementary Figure S27: (a) The running time of scGPCL that leverages MSE-based reconstruction loss over the various number of cells on the simulated data. (b) The running time comparisons of two variants of scGPCL (One that leverages MSE-based reconstruction loss vs. One that leverages ZINB-based reconstruction loss) over the various number of cells on the simulated data.

(a)

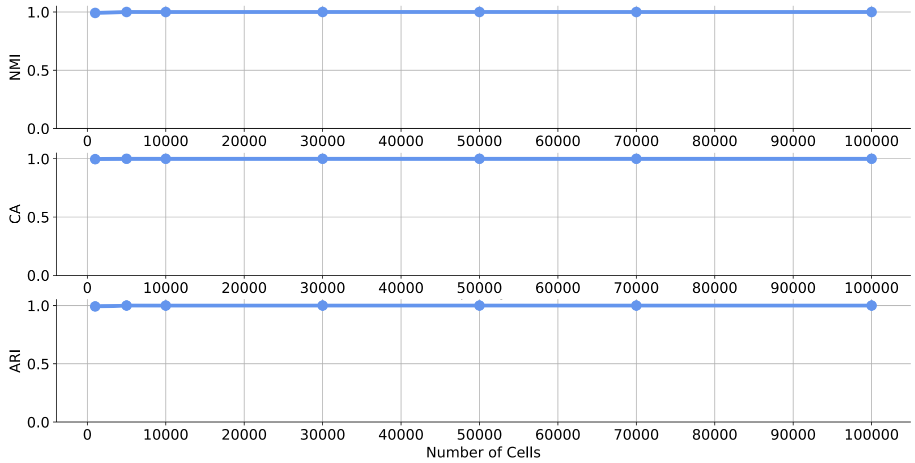

(b)

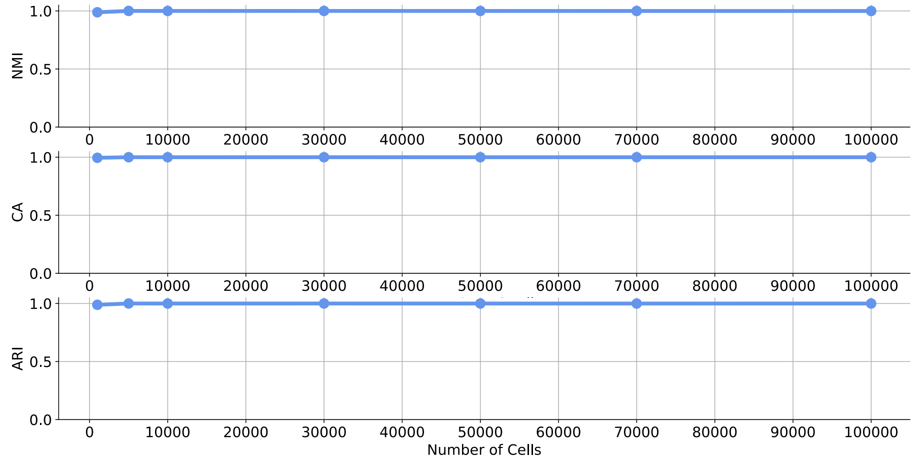

Supplementary Figure S28: Performance of scGPCL as the number of cells increase. (a) shows the performance of original scGPCL, and (b) shows the performance of scGPCL using the MSE based reconstruction loss.

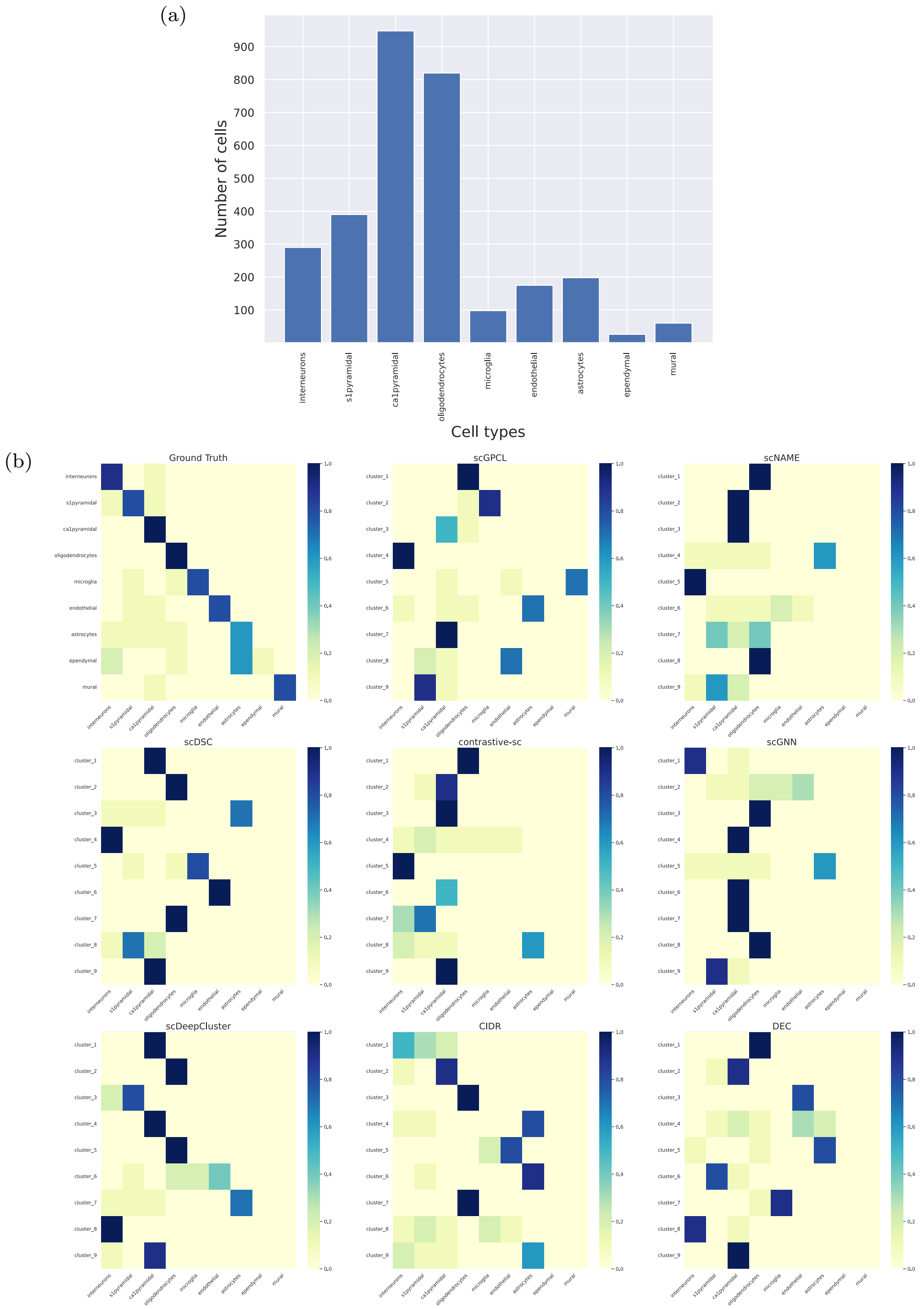

Supplementary Figure S29: Marker gene identification analysis on ‘Zeisel’ dataset. (a) Histogram of cell types and (b) overlap between gold standard cell types and the top 10 DEGs in clusters detected by ground truth cell type, scGPCL, and baseline methods.

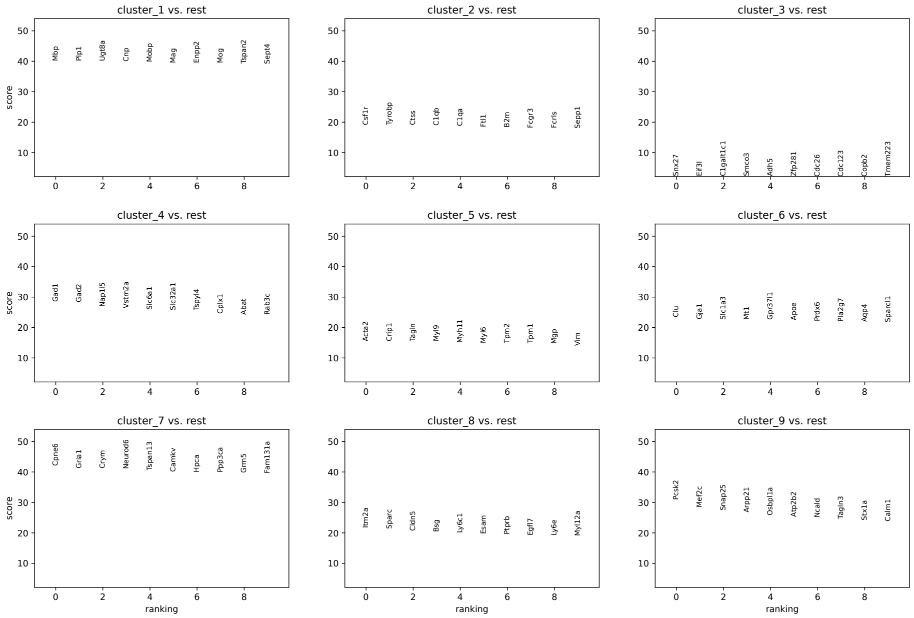

Supplementary Figure S30: Marker gene identification analysis on 'Zeisel' dataset. Ranking of genes in different cell types obtained through the Wilcoxon Rank Sum test by scGPCL.

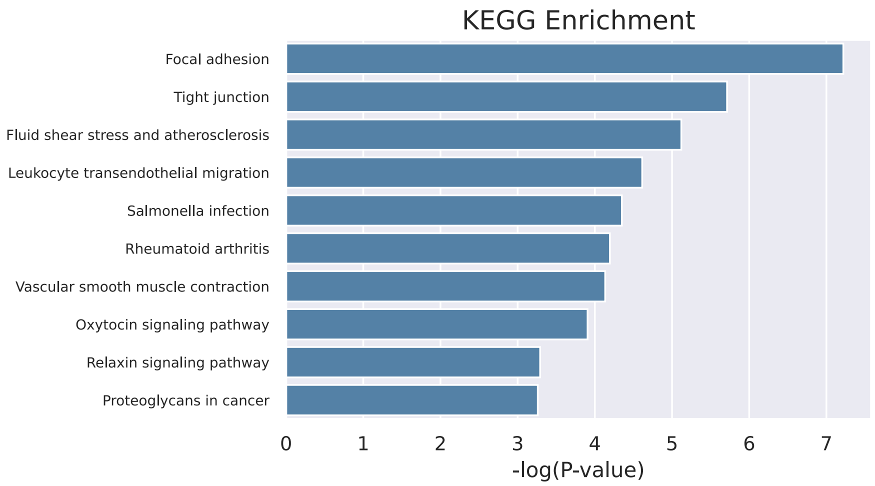

Supplementary Figure S31: Top 10 results of the KEGG enrichment analysis in Zeisel dataset.

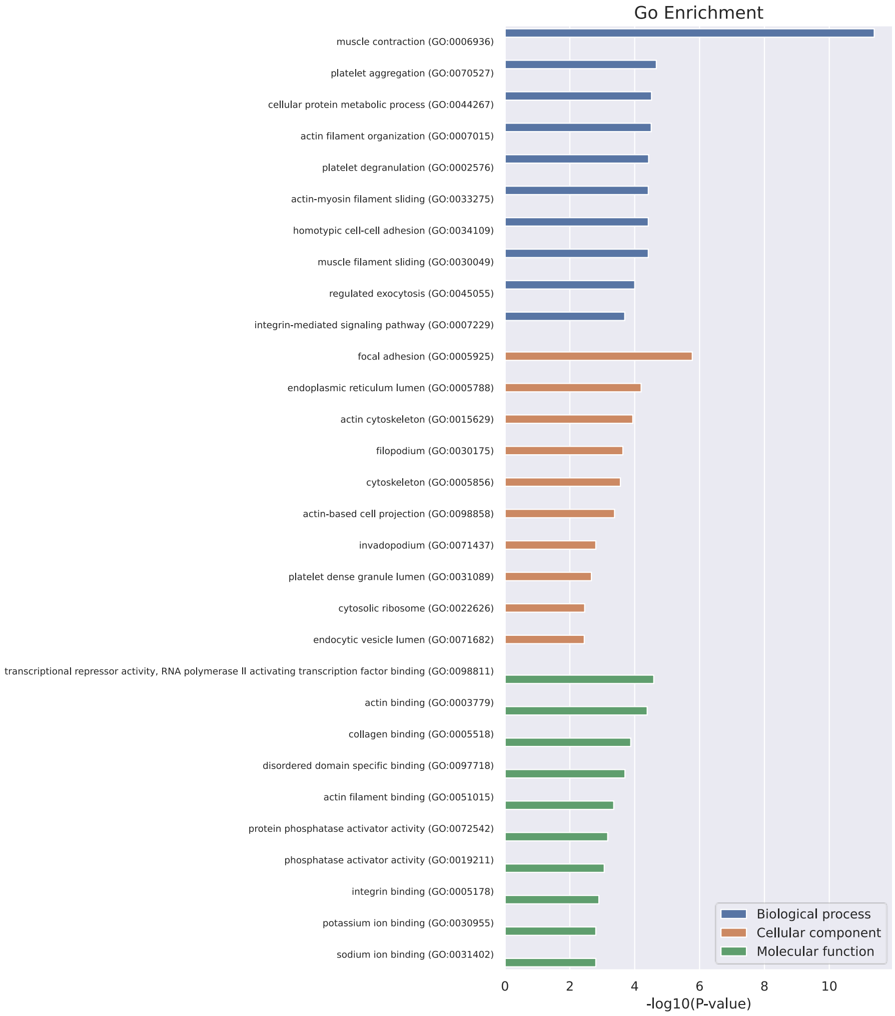

Supplementary Figure S32: Top 10 results of the GO enrichment analysis for Biological process, Cellular component, and Molecular function in the Zeisel dataset.

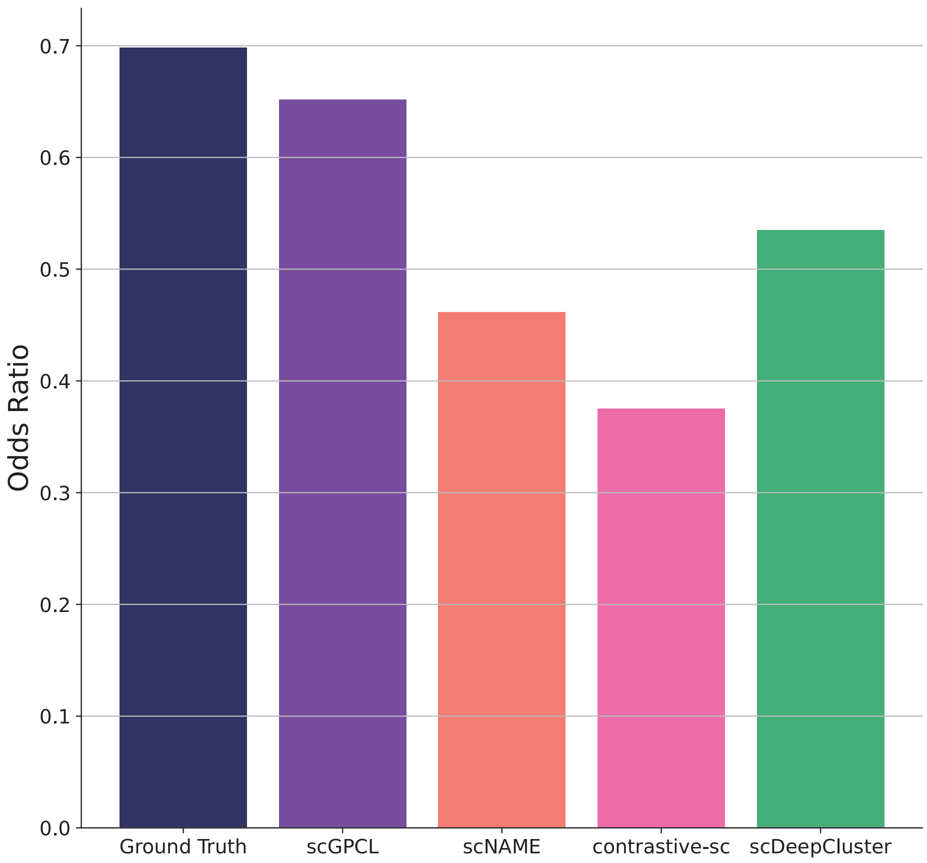

Supplementary Figure S33: Performance comparison of cell-cell communication inference on Mouse Brain Atlas data. The odds ratio represents the ratio of true and false positives among the top 10% ranked interactions versus the same ratios for the remainder of the interactions.

3. Supplementary Tables

Data	Sequencing platform	# of Cells	# of Genes	# of Subgroups
Camp	SMARTer	777	19,020	7
Mouse ES cells	inDrop	2,717	24,047	4
Mouse bladder cells	Microwell-seq	2,746	19,771	16
Zeisel	STRT-seq UMI	3,005	19,972	9
Worm neuron cells	sci-RNA-seq	4,186	13,488	10
10X PBMC	10X	4,340	19,773	8
Human kidney cells	10X	5,685	25,215	11
Baron	inDrop	8,569	20,125	14
Shekhar mouse retina cells	Drop-seq	27,499	13,166	19

Supplementary Table S1: Statistics for real datasets used for experiments.

Method	Language	Reference
scNAME	Python	`https://github.com/aster-ww/scNAME`
scDSC	Python	`https://github.com/DHUDBlab/scDSC`
contrastive-sc	Python	`https://github.com/ciortanmadalina/contrastive-sc`
scGNN	Python	`https://github.com/juexinwang/scGNN`
scDeepCluster	Python	`https://github.com/ttgump/scDeepCluster_pytorch`
CIDR	R	`https://github.com/VCCRI/CIDR`
DEC	Python	`https://github.com/XifengGuo/DEC-keras`

Supplementary Table S2: Link for the source codes of the baseline methods

Dataset	Method						
	scNAME	scDSC	contrastive-sc	scGNN	scDeepCluster	CIDR	DEC
Dropout (3)	0.0000	0.0000	0.0000	0.0000	**0.0704**	0.0000	0.0000
Dropout (6)	0.0000	0.0000	0.0000	0.0000	0.0004	0.0000	0.0000
Dropout (10)	0.0000	0.0000	0.0000	0.000	0.0000	0.0000	0.0000
Signal	0.0000	0.0000	0.0000	0.0000	0.0000	0.0000	0.0000
Imbalance	0.0000	0.0000	0.0000	0.0000	0.0000	0.0000	0.0000

Supplementary Table S3: Statistical significance test on simulated datasets to validate whether the performance of scGPCL is greater than that of baselines in terms of NMI. Specifically, we report the p-value of one-sided paired t-test and bold values represent non-significant results under a significance level of 0.05. In this experiment, Dropout (3), Dropout (6), and Dropout (10) represent the simulated dataset with the highest dropout rate with 3, 6, and 10 clusters, respectively. Signal and Imbalance denote the simulated dataset with the lowest signal and the lowest minimum retention rate, respectively.

Metric	scNAME	scDSC	contrastive-sc	scGNN	scDeepCluster	CIDR	DEC
NMI	0.0137	0.0020	0.0059	0.0020	0.0059	0.0020	0.0020
CA	0.0371	0.0020	0.0059	0.0020	0.0195	0.0020	0.0020
ARI	0.0488	0.0020	0.0059	0.0020	0.0039	0.0020	0.0020

Supplementary Table S4: Statistical significance test over all the nine datasets to validate whether the performance of scGPCL is greater than that of baselines in terms of NMI, CA, and ARI. Specifically, we report the p-value of one-sided Wilcoxon singed rank tests between scGPCL and baseline methods.

Dataset	Method						
	scNAME	scDSC	contrastive-sc	scGNN	scDeepCluster	CIDR	DEC
Camp	0.0017	**0.1433**	0.0018	**0.3593**	0.0051	0.0000	0.0008
Mouse ES cells	0.0004	0.0000	0.0000	0.0045	0.0000	0.0000	0.0000
Mouse bladder cells	0.0018	0.0000	0.0004	0.000	0.000	0.000	0.000
Zeisel	0.0000	0.0000	0.0000	0.0000	0.0000	0.0000	0.0000
Worm neuron cells	0.0000	0.0000	0.0001	0.0000	0.0000	0.0000	0.0000
10X PBMC	**0.0691**	0.0000	0.0000	0.0000	**0.0976**	0.0000	0.0000
Human kidney cells	0.0001	0.0000	0.0000	0.0000	0.0000	0.0000	0.0000
Baron	0.0000	0.0000	0.0000	0.0000	0.0000	0.0000	0.0000
Shekhar mouse retina cells	0.0048	0.0000	0.0000	0.0000	0.0013	0.0000	0.0000

Supplementary Table S5: Statistical significance test on real datasets to validate whether the performance of scGPCL is significantly greater than that of the baselines in terms of NMI. We report the p-value of one-sided paired t-test. More precisely, when scGPCL is not the best performing model, we define the null hypothesis as $H_0 : NMI_{\text{baseline}} > NMI_{\text{scGPCL}}$, and when scGPCL is the best performing model, we define it as $H_0 : NMI_{\text{scGPCL}} > NMI_{\text{baseline}}$. Here bold values represent non-significant results under a significance level of 0.05.

4. Supplementary Algorithms

Algorithm S1 Algorithm for the pre-training phase of scGPCL.

for epoch $\leftarrow 1, 2, \ldots,$ maximum epoch **do**
  for batch $\leftarrow 1, 2, \ldots$ **do**
    Generate two augmented graphs $\tilde{\mathcal{G}}$ and $\tilde{\mathcal{G}}'$ using stochastic augmentation with respect to the anchor nodes
    Obtain cell representations for anchor nodes: $\tilde{H} \leftarrow f(\tilde{\mathcal{G}}), \tilde{H}' \leftarrow f(\tilde{\mathcal{G}}')$ (Eqn. 1)
    Calculate the instance-wise contrastive loss $\mathcal{L}_{\text{Ins}}$ using $\tilde{H}$ and $\tilde{H}'$ (Eqn. 3)
    Obtain $\tilde{M}, \tilde{\Theta}, \tilde{\Pi}$ from $\tilde{H}$ and $\tilde{M}', \tilde{\Theta}', \tilde{\Pi}'$ from $\tilde{H}'$ (Eqn. 7)
    Calculate the ZINB-based reconstruction loss $\mathcal{L}_{\text{ZINB}}^{\text{Pre}}$ using $\tilde{M}, \tilde{\Theta}, \tilde{\Pi}, \tilde{M}', \tilde{\Theta}', \tilde{\Pi}'$ (Eqn. 9)
    if epoch $>$ warm-up epoch **then**
      $Z_{\text{set}} \leftarrow$ ASSIGN_PROTOTYPE($\tilde{H}'$)
      Calculate the prototypical contrastive loss $\mathcal{L}_{\text{Pro}}$ using $\tilde{H}$ and Z_{set} (Eqn. 5)
    end if
    Update parameters using $\mathcal{L}_{\text{Pre}}$ (Eqn. 10)
  end for
  if epoch $>$ warm-up epoch **then**
    Obtain cell representations $\hat{H}$ for all cells in $\mathcal{G}$ using GNN encoder f (i.e., $\hat{H} = f(\mathcal{G})$)
    $Y_{\text{new}} \leftarrow K\text{-}means(\hat{H})$
    if $ARI(Y_{new}, Y_{old}) > r$ **then**
      break
    else
      $Y_{old} \leftarrow Y_{new}$
    end if
  end if
end for

function ASSIGN_PROTOTYPE(H)
  $Z_{\text{set}} \leftarrow []$
  for $K \leftarrow K^1, K^2, \ldots, K^T$ **do**
    Cluster each cell into K clusters based on H
    Obtain a prototype matrix $Z \in \mathbb{R}^{K \times d}$ by computing the average of the cell representations that belong to each cluster
    Append Z to Z_{set}
  end for
end function

Algorithm S2 Algorithm for the fine-tuning phase of scGPCL.

for epoch $\leftarrow 1, 2, \dots$, maximum fine-tuning epoch **do**
  Obtain cell representations for all cells in $\mathcal{G}$ using GNN encoder f (i.e., $\hat{H} = f(\mathcal{G})$)
  Compute the soft cluster assignment $\hat{Q}$ using $\hat{H}$ (Eqn. 12)
  Compute the target distribution $\hat{P}$ using $\hat{Q}$ (Eqn. 13)
  if $\frac{Y_{new} \neq Y_{old}}{N_c} < tol\%$ **then**
    break
  else
    $Y_{old} \leftarrow Y_{new}$
  end if
  for batch $\leftarrow 1, 2, \dots$ **do**
    Augment the original Graph $\mathcal{G}$ into $\tilde{\mathcal{G}}$ using stochastic augmentation
    Obtain anchor cell representation $\tilde{H}$ of $\tilde{\mathcal{G}}$ using GNN encoder f (Eqn. 1)
    Obtain $\tilde{M}, \tilde{\Theta}, \tilde{\Pi}$ from $\tilde{H}$ (Eqn. 7)
    Calculate the ZINB-based reconstruction loss $\mathcal{L}_{\mathsf{ZINB}}^{\mathsf{Fine}}$ using $\tilde{M}, \tilde{\Theta}, \tilde{\Pi}$ (Eqn. 15)
    Obtain Q using $\tilde{H}$
    Obtain P by extracting rows from $\hat{P}$ corresponding to the anchor nodes in the current batch.
    Calculate the clustering task oriented loss $\mathcal{L}_{\mathsf{Cluster}}$ using Q and P (Eqn. 11)
  end for
  Update parameters using $\mathcal{L}_{\mathsf{Fine}}$ (Eqn. 14)
end for

References

[1] Baron, M. *et al.* (2016). A single-cell transcriptomic map of the human and mouse pancreas reveals inter-and intra-cell population structure. *Cell systems*, **3**(4), 346–360.

[2] Bla Bland, J. (2000). Statistics notes: the odds ratio. *Bmj*, **320**(7247), 1468.

[3] Camp, J. G. *et al.* (2017). Multilineage communication regulates human liver bud development from pluripotency. *Nature*, **546**(7659), 533–538.

[4] Cao, J. *et al.* (2017). Comprehensive single-cell transcriptional profiling of a multicellular organism. *Science*, **357**(6352), 661–667.

[5] Chen, T.-S. *et al.* (2021). Incremental false negative detection for contrastive learning. *arXiv preprint arXiv:2106.03719*.

[6] Ciortan, M. and Defrance, M. (2021). Contrastive self-supervised clustering of scrna-seq data. *BMC bioinformatics*, **22**(1), 1–27.

[7] Dimitrov, D. *et al.* (2022). Comparison of methods and resources for cell-cell communication inference from single-cell rna-seq data. *Nature Communications*, **13**(1), 3224.

[8] Efremova, M. *et al.* (2020). Cellphonedb: inferring cell–cell communication from combined expression of multi-subunit ligand–receptor complexes. *Nature protocols*, **15**(4), 1484–1506.

[9] Eraslan, G. *et al.* (2019). Single-cell rna-seq denoising using a deep count autoencoder. *Nature communications*, **10**(1), 1–14.

[10] Foo, S. S. *et al.* (2006). Ephrin-b2 controls cell motility and adhesion during blood-vessel-wall assembly. *Cell*, **124**(1), 161–173.

[11] Gan, Y. *et al.* (2022). Deep structural clustering for single-cell rna-seq data jointly through autoencoder and graph neural network. *Briefings in Bioinformatics*, **23**(2), bbac018.

[12] Hamilton, W. *et al.* (2017). Inductive representation learning on large graphs. *Advances in neural information processing systems*, **30**.

[13] Han, X. *et al.* (2018). Mapping the mouse cell atlas by microwell-seq. *Cell*, **172**(5), 1091–1107.

[14] Huynh, T. *et al.* (2022). Boosting contrastive self-supervised learning with false negative cancellation. In *Proceedings of the IEEE/CVF Winter Conference on Applications of Computer Vision*, pages 2785–2795.

[15] Khosla, P. *et al.* (2020). Supervised contrastive learning. *Advances in Neural Information Processing Systems*, **33**, 18661–18673.

[16] Klein, A. M. *et al.* (2015). Droplet barcoding for single-cell transcriptomics applied to embryonic stem cells. *Cell*, **161**(5), 1187–1201.

[17] Lin, P. *et al.* (2017). Cidr: Ultrafast and accurate clustering through imputation for single-cell rna-seq data. *Genome biology*, **18**(1), 1–11.

[18] Loshchilov, I. and Hutter, F. (2017). Decoupled weight decay regularization. *arXiv preprint arXiv:1711.05101*.

[19] Luecken, M. D. and Theis, F. J. (2019). Current best practices in single-cell rna-seq analysis: a tutorial. *Molecular systems biology*, **15**(6), e8746.

[20] Shekhar, K. *et al.* (2016). Comprehensive classification of retinal bipolar neurons by single-cell transcriptomics. *Cell*, **166**(5), 1308–1323.

[21] Tasic, B. *et al.* (2016). Adult mouse cortical cell taxonomy revealed by single cell transcriptomics. *Nature neuroscience*, **19**(2), 335–346.

[22] Tian, T. *et al.* (2019). Clustering single-cell rna-seq data with a model-based deep learning approach. *Nature Machine Intelligence*, **1**(4), 191–198.

[23] Tian, T. *et al.* (2021). Model-based deep embedding for constrained clustering analysis of single cell rna-seq data. *Nature communications*, **12**(1), 1–12.

[24] Wan, H. *et al.* (2022). scname: neighborhood contrastive clustering with ancillary mask estimation for scrna-seq data. *Bioinformatics*, **38**(6), 1575–1583.

[25] Wang, J. *et al.* (2021). scgnn is a novel graph neural network framework for single-cell rna-seq analyses. *Nature communications*, **12**(1), 1–11.

[26] Xie, J. *et al.* (2016). Unsupervised deep embedding for clustering analysis. In *International conference on machine learning*, pages 478–487. PMLR.

[27] Young, M. D. *et al.* (2018). Single-cell transcriptomes from human kidneys reveal the cellular identity of renal tumors. *science*, **361**(6402), 594–599.

[28] Zeisel, A. *et al.* (2015). Cell types in the mouse cortex and hippocampus revealed by single-cell rna-seq. *Science*, **347**(6226), 1138–1142.

[29] Zheng, G. X. *et al.* (2017). Massively parallel digital transcriptional profiling of single cells. *Nature communications*, **8**(1), 1–12.
